# Evolutionary history of selenocysteine incorporation from the perspective of SECIS binding proteins

**DOI:** 10.1186/1471-2148-9-229

**Published:** 2009-09-10

**Authors:** Jesse Donovan, Paul R Copeland

**Affiliations:** 1 Department of Molecular Genetics, Microbiology, and Immunology, Graduate School of Biomedical Sciences, University of Medicine and Dentistry of New Jersey - Robert Wood Johnson Medical School, 675 Hoes Lane, Piscataway, NJ, USA

## Abstract

**Background:**

The co-translational incorporation of selenocysteine into nascent polypeptides by recoding the UGA stop codon occurs in all domains of life. In eukaryotes, this event requires at least three specific factors: SECIS binding protein 2 (SBP2), a specific translation elongation factor (eEFSec), selenocysteinyl tRNA, and a *cis*-acting selenocysteine insertion sequence (SECIS) element in selenoprotein mRNAs. While the phylogenetic relationships of selenoprotein families and the evolution of selenocysteine usage are well documented, the evolutionary history of SECIS binding proteins has not been explored.

**Results:**

In this report we present a phylogeny of the eukaryotic SECIS binding protein family which includes SBP2 and a related protein we herein term SBP2L. Here we show that SBP2L is an SBP2 paralogue in vertebrates and is the only form of SECIS binding protein in invertebrate deuterostomes, suggesting a key role in Sec incorporation in these organisms, but an SBP2/SBP2L fusion protein is unable to support Sec incorporation *in vitro*. An in-depth phylogenetic analysis of the conserved L7Ae RNA binding domain suggests an ancestral relationship with ribosomal protein L30. In addition, we describe the emergence of a motif upstream of the SBP2 RNA binding domain that shares significant similarity with a motif within the pseudouridine synthase Cbf5.

**Conclusion:**

Our analysis suggests that SECIS binding proteins arose once in evolution but diverged significantly in multiple lineages. In addition, likely due to a gene duplication event in the early vertebrate lineage, SBP2 and SBP2L are paralogous in vertebrates.

## Background

All domains of life possess the ability to recode select UGA codons from a translation termination signal to a selenocysteine (Sec) codon. The translation products of successful Sec incorporation are termed selenoproteins. In eukaryotes the recognition of UGA as a Sec codon by the protein synthetic machinery requires a *cis*-acting Sec insertion sequence (SECIS) element in the 3' untranslated regions (UTRs) of selenoprotein mRNAs. Eukaryotic SECIS elements are stable stem-loop structures that are comprised of two helices separated by a kink-turn containing a conserved GA quartet (SECIS core) and an apical AAR motif that is present either as a terminal loop (Form 1) or a terminal bulge (Form 2) [reviewed in [1]]. The *trans*-acting factors known to be required for Sec incorporation are SECIS binding protein 2 (SBP2), Sec-tRNA^Sec^, and the Sec specific translation elongation factor, eEFSec. SBP2 is the most studied of the known *trans*-acting eukaryotic Sec incorporation factors. Structure/function studies of rat SBP2 have delineated three domains: a putative regulatory domain comprising the N-terminal half of the protein (aa 1-407; human numbering is used throughout unless otherwise noted), a Sec incorporation domain (SID; aa 408-545) and an RNA binding domain (RBD, aa 623-784) [2,3]. The SBP2 RBD contains an L7Ae motif that binds the kink-turn structure found in SECIS RNA [4,5]. While the RBD is capable of interacting with SECIS elements alone, its affinity is greatly enhanced by the SID. An SBP2/SECIS complex recruits eEFSec and a model was proposed that in the SBP2/SECIS/eEFSec complex the SID is conformationally competent to prime the ribosome for Sec incorporation [2].

Previous studies have noted the existence of an apparent SBP2 paralogue, termed KIAA0256 in sequence databases and referred to in the literature as SBP2-like protein [3,6]. Current NCBI annotations for KIAA0256 have adopted the nomenclature SECISBP2L, which we abbreviate here as SBP2L. SBP2L has an L7Ae RNA binding motif and a domain with clear sequence homology to the SID. Despite the similarity to SBP2, the C-terminal fragment of human SBP2L (aa 467-1101) that is analogous to the fully functional C-terminal fragment of SBP2 (aa 408-854) was not competent for Sec incorporation *in vitro *and displayed only minimal SECIS binding [3].

The evolutionary histories of selenoprotein families have been well documented, but to date SBP2 phylogeny has only been analyzed in insects [7]. As such we sought to gather more evolutionary insight into Sec incorporation by undertaking a phylogenetic analysis of SBP2 as well as a study of its relationship to SBP2L. Based on genome analysis, we speculate that mammalian SBP2L may play a role in selenoprotein expression but we also show that an SBP2L/SBP2 chimeric protein is unable to promote Sec incorporation *in vitro*. Additionally, we trace the lineage of the SBP2/SBP2L RNA binding domain to archaeal ribosomal protein L30.

## Results and discussion

### SBP2L and SBP2 diverged during vertebrate evolution

**Figure 1:**
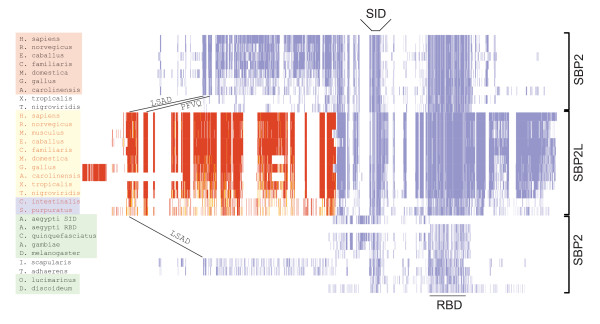
**SECIS binding protein topology**. Alignment of SBP2 and SBP2L across the species indicated generated with the MUSCLE module in Geneious (Biomatters Ltd). The N and C-terminal portions of SBP2 and SBP2L were independently aligned. Residues were colored using JalView based on BLOSUM62 score [[37]; darker colors indicate higher similarity]. The SBP2L N-terminal sequence is red to denote that it is a separate alignment from that for SBP2. Globally conserved motifs motifs include the Sec incorporation domain (SID), the RNA binding domain (RBD), LSAD^15-26 ^and PFVQ^44-56^. Shading of species names is used for the identification of SBP2 classes as described in the text.

The occurrence of SBP2 and SBP2L among eukaryotes can be divided into three major categories. Figure 1 provides a representation of SBP2/SBP2L protein topology as a global sequence alignment that includes examples of SBP2 and its homologues from a broad sampling of species that are known to express selenoproteins. The first category is found in most vertebrates (red shading), which possess a version of SBP2 that contains a ~400 amino acid N-terminal extension upstream of two domains known to be required for Sec incorporation: the Sec incorporation domain (SID) and the SECIS RNA binding domain (RBD). In addition to SBP2, vertebrates also possess the aforementioned SBP2-like protein, SBP2L (yellow shading), which also contains an N-terminal extension that is only partially related to that found in SBP2. The second category includes most protostomes as well as unicellular green algae and slime molds (Figure 1, green shading), which possess a version of SBP2 that lacks the N-terminal extension found in mammals. Lastly, the third category consists of invertebrate deuterostomes (violet shading), which possess only SBP2L. While all of the organisms studied for this report contain sequences related to the SID and RBD, the N-terminal portion of SBP2/SBP2L is highly variable as exemplified by the versions of SBP2 found in frog (*Xenopus tropicalis*), fish (*Tetraodon nigroviridis*), black legged tick (*Ixodes scapularis*) and the placazoan, *Trichoplax adhaerens*.

**Figure 2:**
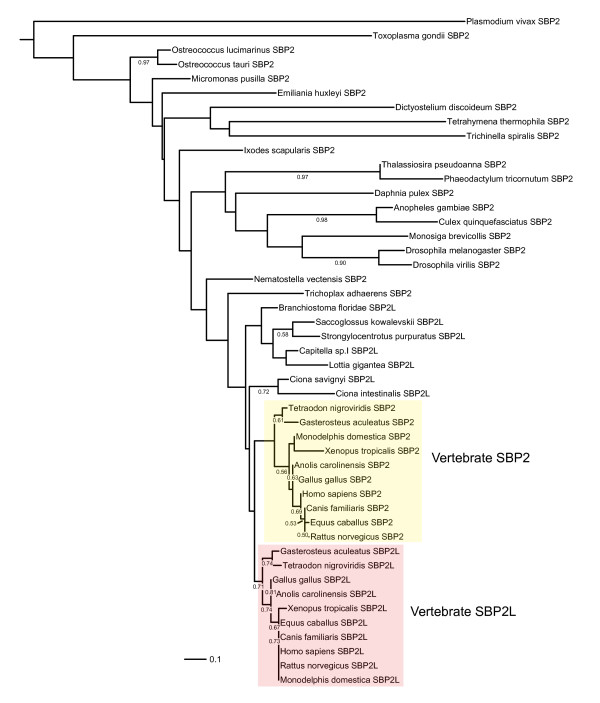
**Phylogeny of SECIS binding proteins across eukaryotic taxa**. SECIS binding proteins from the indicated taxa were aligned with MUSCLE and non-conserved regions were removed with GBlocks. The resulting alignment was used to infer a maximum likelihood tree with PhyML with 500 bootstrap replicates. Bootstrap values less than 0.5 are not shown.

In order to determine if the core conserved regions of SBP2 and SBP2L are phylogenetically distinguishable, we performed a global sequence alignment of the C-terminal portions of both proteins. We obtained full length sequences either from the NCBI Refseq database or genomic sequence from a broad taxonomic sampling ranging from unicellular eukaryotes to mammals. Sequences derived from genomic data were compiled by gene prediction using FgenesH [8] and/or Genomescan [9]. Only sequences that could be validated with expressed sequence tag (EST) data were used. A global sequence alignment containing the entirety of all 47 sequences was generated using the MUSCLE algorithm. For the purposes of phylogenetic analysis, non-conserved sequences were eliminated using GBlocks leaving only the highly conserved regions in the SID (G518-K529) and RBD (K639-G754). Figure 2 shows a phylogenetic tree based on maximum likelihood using the Whelan & Goldman substitution model in PhyML [[10,11]; see Additional file 1 for raw sequence alignment data]. The resulting tree illustrates the segregation of vertebrate SBP2 and SBP2L into separate sub-clades restricting the occurrence of SBP2L to all deuterostomes, the mollusk (*Lottia gigantea*), and an annelid worm (*Capitella sp.I*). In addition, Figure 2 shows that SBP2L from the invertebrate deuterostomes (*Strongylocentrotus purpuratus, Branchiostoma floridae, Ciona savignyi, Ciona intestinalis*, and *Saccoglossus kowalevskii*), a mollusk *L. gigantia*, and the annelid worm *C. sp.I *are in a separate clade from that of vertebrates. Despite this distinction we maintain that the SBPs in invertebrate deuterostomes, *L. gigantia *and *C. sp.I *are SBP2L due to the presence of conserved motifs found in vertebrate SBP2L but not SBP2 (see below). Since the only form of a SECIS binding protein in these organisms (see below), which are known to possess selenoprotein genes, is SBP2L, it is highly probable that SBP2L is carrying out the functions of SBP2 in these cases. These results indicate that even when only the highly conserved SID and RBD regions are considered, SBP2 and SBP2L are phylogenetically distinct.

**Figure 3:**
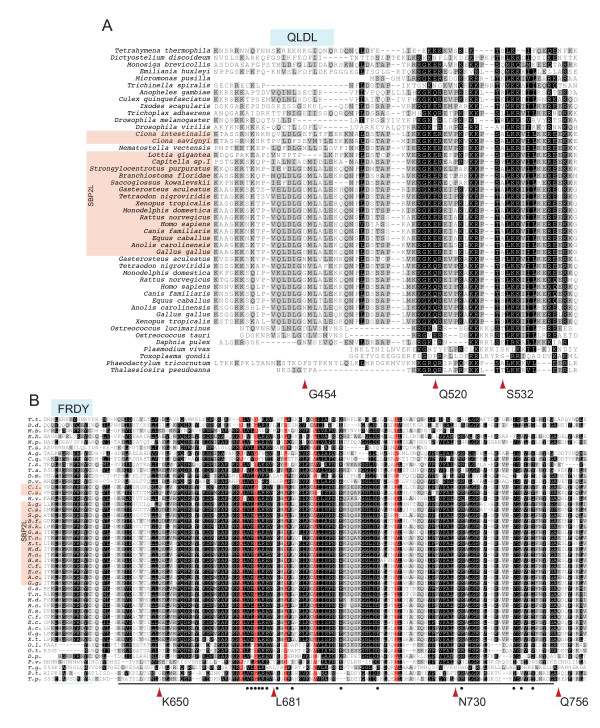
**The SBP SID and RBD are highly conserved across Eukarya**. (A) An extraction of the SBP2 and SBP2L SID region from the global multiple sequence alignment described for Figure 2 prior to GBlocks treatment. The non-conserved region between 464-505 (human SBP2 numbering) was deleted. (B) An extraction of the SBP2 and SBP2L RBD region as in (A). Nonconserved sequences from *M. brevicollis*, *E. huxleyi*, and *T. gondii *that introduced large gaps in the region were deleted. In addition the sequence from *S. purpuratus *was omitted from this alignment due the presence of several large non-conserved regions that disrupted the entire global alignment. Positions that are consistently variable between SBP2 and SBP2L are indicated with red arrows and the identity/number refers to the position in human SBP2. Underlined sequence corresponds to the GBlocks output that was used to generate the tree in Figure 2. Blue shaded boxes indicate conserved motifs that are only sporadically found in unicellular organisms.

In order to examine the specific features that discriminate between SBP2 and SBP2L within the conserved SID and RBD, we examined an expanded multiple sequence alignment that includes the regions used to generate the tree in Figure 2. Within these highly conserved regions, there are individual residues that are found predominantly in either SBP2L or SBP2 (positions marked with an arrow, Figure 3A and 3B). These include G454, Q520 and S532 (human SBP2 numbering) in the SID and K650, L681, N730 and Q756 in the RBD. Considering the proximity of these residues to those known to be required for Sec incorporation (Q520), ribosome binding (D454) and SECIS binding (A532, K650, L681 and N730) in SBP2 [2,12,13], it is likely that some of these residues may be required for sequence specific RNA binding in the case of the RBD and specific integration of an RBD-dependent signal (i.e. eEFSec or ribosome conformation) in the case of the SID. The alignment shown in Figure 3 also serves to highlight two sequence motifs that are conserved among deuterostomes but also in a mosaic of protostomes and unicellular eukaryotes (QLDL^449-452 ^and FRDY^629-632^). Conserved motifs are designated as the human SBP2 or SBP2L sequence corresponding to the four most highly conserved residues in the motif with numbers indicating the full range of conserved sequence. If a motif is found in both SBP2 and SBP2L, the SBP2 numbering is used. The QLDL^449-452 ^motif is found in the cnidarian *Nematostella vectensis *but not in the protostome *Drosophila melanogaster*. On the other hand it is found in the mosquito *Anopheles gambiae *but not in *Dictyostelium discoideum*. In contrast, FRDY^629-632 ^is found in all protostomes surveyed, but among unicellular eukaryotes and other metazoans representing ancient lineages it is only present in *D. discoideum, Monosiga brevicollis, Emiliania huxleyi *and *T. adhaerens*. These results suggest that both of these motifs were present in the last common ancestor of Sec-utilizing eukaryotes but that they were lost during the evolution of select lineages, likely due to specific constraints (or relief of constraints) at the level of the SECIS element or ribosome.

**Figure 4:**
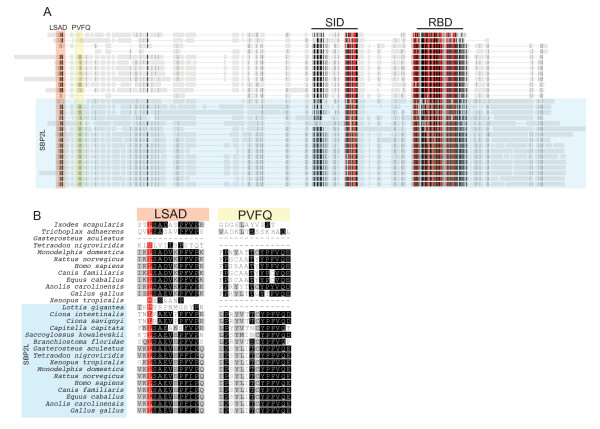
**Identification of conserved motifs common to SBP2 and SBP2L**. (A) Global Alignment of complete sequences for SBP2L and vertebrate SBP2 from the taxa indicated in (B) generated with MUSCLE. Conserved residues are colored according to Blosum62 score with red denoting 100% conservation, black 80-99% identical, dark grey 60-80% identical, light grey less than 60% identical. Conserved motifs are shaded by colored boxes. (B) Conserved motifs in SBP2L and SBP2 marked by colored boxes in panel A are shown in detail.

Biochemical characterization of SBP2L has been limited, but early tests showed that the C-terminal portion of the protein, which is ~45% identical to the fully functional C-terminal SBP2 counterpart in humans, was unable to support Sec incorporation *in vitro*. This correlated with the fact that SBP2L only weakly bound the GPX4 SECIS that was used in this assay [3]. These preliminary biochemical analyses, together with the fact that SBP2L is the only form of SBP in invertebrate deuterostomes (see below) as well as the fact that it is not found in organisms that do not express selenoproteins (e.g. higher plants and fungi; data not shown), suggest that SBP2L is involved in selenoprotein synthesis. To gain further insight into the potential function for SBP2L and the evolutionary origins of SECIS binding proteins, we first delineated the major features that distinguish SBP2L from SBP2. SBP2L is consistently larger in size than SBP2 as the average predicted molecular weight of chicken, rat, human, dog, and horse SBP2L is 126 kDa while that of SBP2 from the same species is 98 kDa. In terms of sequence similarity, several important differences are notable. Figure 4 illustrates a global sequence alignment containing vertebrate SBP2, SBP2L from all of the 17 species that we have identified as possessing complete SBP2L sequence, and unique variants of SBP2 from *I. scapularis *and *T. adhaerens*. The method used for identifying SBP2 and SBP2L sequences as well as the complete amino acid sequences and accession numbers used for this study are found in Additional files 2 and 3 respectively. Significant sequence similarity between SBP2 and SBP2L is limited to the regions comprising the SID and RBD. The N-terminal portions are much more divergent with the exception of two small motifs present at the N-terminus of both SBP2L and SBP2: LSAD^15-26 ^and PFVQ^44-56^, the former of which has been previously noted [14]. These motifs are separated by degenerate sequence of variable length, generally 29-30 residues in SBP2L and 18 residues in SBP2. A detailed sequence alignment of this region is shown in Figure 4B. Interestingly, we found that an arthropod (the tick *I. scapularis*) and the placozoan, *T. adhaerens *(considered by some to be representative of the most ancient metazoans [15]) also contain an N-terminal LSAD^15-26 ^but not the PFVQ^44-56 ^motif, thus representing exceptions to the apparent restriction of the LSAD^15-26 ^motif to deuterostomes. It is also noteworthy that SBP2 from frog (*X. tropicalis*) and fish (*T. nigroviridis*) lacks both of these motifs yet they are present in their respective versions of SBP2L. Since we have identified both of these motifs in lizard (*Anolis carolinensis*) SBP2, we speculate that the LSAD^15-26 ^and PFVQ^44-56 ^motifs were lost in the lineages giving rise to fish and amphibians. In addition, Papp et al [16] recently identified several splice variants of human SBP2, the most abundant of which encodes a mitochondrial targeted protein (mtSBP2). Interestingly, this splice isoform lacks both the LSAD^15-26 ^and PFVQ^44-56 ^motifs but retains the SBP2-specific motifs presented below. Additionally, the authors suggested that mtSBP2 may be unique to humans based on comparison to other mammals and chicken.

**Figure 5:**
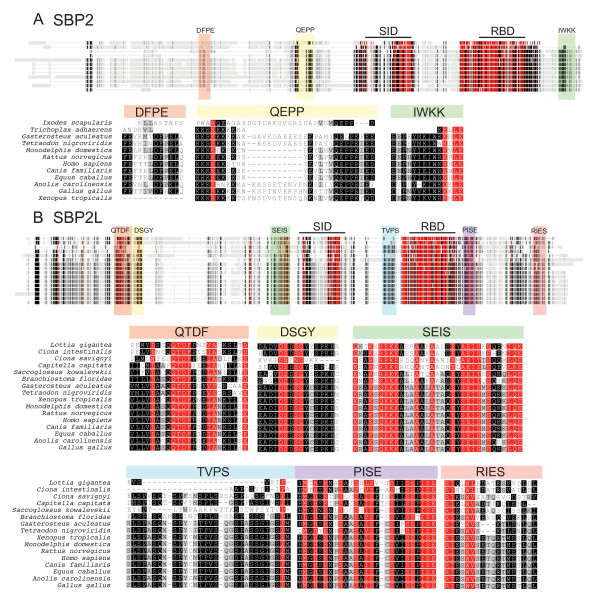
**Identification of conserved motifs within SBP2 and SBP2L**. (A) Global alignment of vertebrate SBP2 sequences (top) with detailed alignment of conserved motifs as indicated (bottom). (B) Global and detailed alignments of SBP2L as in (A).

SBP2L and SBP2 are further distinguished by the presence of highly conserved sequence motifs that are unique to each protein. Figure 5 highlights these motifs in separate SBP2 and SBP2L multiple sequence alignments derived from the same species examined in Figure 4. In SBP2L, the conserved motifs include QTDF^210-252^, DSGY^265-279^, SEIS^442-474^, TPVS^622-662^, PISE^844-868 ^and RIES^1016-1032^. In contrast there are only three sites of SBP2-specific conservation: DFPE^216-226^, QEPP^380-405^, which includes the poly-lysine tract that has been shown to be active as a nuclear localization signal [17], and IWKK^816-839^. Table 1 provides a summary of the occurrence of thee motifs in each species analyzed. Interestingly, none of these regions are annotated in the NCBI Conserved Domains Database, and PSI-BLAST analysis does not link them to any other protein families (data not shown), but the significant degree of identity across highly diverse species makes them important focal points for further investigation into the function and regulation of SBP2 and SBP2L. As shown in Figure 5 and Table 1, we observed variability in the occurrence of some of the motifs, notably the lack of DFPE^216-226 ^in frog and the lack of TPVS^622-662 ^in the sea squirts (*C. intestinalis and C. savignyi*) as well as a the hemichordate acorn worm (*Saccoglossus kowalevskii*). These results confirm the phylogeny shown in Figure 2 demonstrating that SBP2L is not limited to deuterostomes since we identified SBP2L motifs in a mollusk (*L. gigantea*), and an annelid worm (*Capitella sp.I*). Among the conserved motifs in SBP2L, TPVS^622-662 ^is of interest because it lies between the SID and RBD, and the corresponding sequence in SBP2 is degenerate and has been found to be dispensable for Sec incorporation activity [18]. Considering that a dynamic interaction between the SID and RBD has been shown to be essential for Sec incorporation [2], it is likely that the interaction of the corresponding domains in SBP2L may be regulated by this conserved stretch of amino acids.

**Table 1:** Distribution of SBP2 and SBP2L motifs across species

**SBP2**	***LSAD***	***PFVQ***	***DFPE***	***QEPP***	***IWKK***	**SBP2L**	***LSAD***	***PFVQ***	***QQTD***	***DSGY***	***SEIS***	***TPVS***	***EPIS***
*I. scapularis*	✔					*C. intestinalis*	✔	✔	✔	✔	✔		✔

*G. aculeatus*			✔	✔	✔	*C. savignyi*	✔	✔	✔	✔	✔		✔

*T. nigroviridis*			✔	✔	✔	*C. capitata*	✔	✔	✔	✔	✔	✔	✔

*X. tropicalis*				✔	✔	*L. gigantea*			✔		✔	✔	✔

*M. domestica*	✔	✔	✔	✔	✔	*B. floridae*	✔	✔	✔	✔	✔	✔	✔

*R. norvegicus*	✔	✔	✔	✔	✔	*S. kowalevskii*	✔	✔	✔	✔	✔		✔

*C. familiaris*	✔	✔	✔	✔	✔	*S. purpuratus*	✔		✔	✔	✔	✔	✔

*H. sapiens*	✔	✔	✔	✔	✔	*G. aculeatus*	✔	✔	✔	✔	✔	✔	✔

*E. caballus*	✔	✔	✔	✔	✔	*T. nigroviridis*	✔	✔	✔	✔	✔	✔	✔

*A. carolinensis*	✔	✔	✔	✔	✔	*X. tropicalis*	✔	✔	✔	✔	✔	✔	✔

*G. gallus*	✔	✔	✔	✔	✔	*M. domestica*	✔	✔	✔	✔	✔	✔	✔

*T. adhaerens*	✔					*R. norvegicus*	✔	✔	✔	✔	✔	✔	✔

						*H. sapiens*	✔	✔	✔	✔	✔	✔	✔

						*C. familiaris*	✔	✔	✔	✔	✔	✔	✔

						*E. caballus*	✔	✔	✔	✔	✔	✔	✔

						*A. carolinensis*	✔	✔	✔	✔	✔	✔	✔

						*G. gallus*	✔	✔	✔	✔	✔	✔	✔

### Origins of the SBP2/SBP2L split

BLAST and BLAT searches against the genomes of *Lottia*, *Capitella*, and invertebrate deuterostomes (*S. purpuratus*, *Ciona savignyi*, and *C. intestinalis*, and *Saccoglossus kowalevskii*) revealed that SBP2L is the only SECIS binding protein in these organisms. All vertebrate genomes sampled, however, encoded both SBP2 and SBP2L, suggesting that a gene duplication near the time of vertebrate emergence generated the paralogous SBP2/SBP2L pair. The paralogy of SBP2 and SBP2L in vertebrates is supported by their inferred phylogeny (Figure 2).

The 2R hypothesis posits that one round of whole genome duplication coincided with the divergence of vertebrates and urochordates (e.g. *Ciona *species) and a second round of whole genome duplication occurred with the divergence of jawless (lamprey and hagfish) and jawed vertebrates [19]. In the context of the 2R hypothesis, the time of SBP2/SBP2L divergence is uncertain. Therefore, we sought to find SBP sequences in the sea lamprey (*Petromyzon marinus*) genome by BLAT search. We found two non-overlapping contigs (contigs 9804 and 33761) that contain parts of an RBD and SID, respectively. However, there does not appear to be sufficient sequence available to determine whether or not the sea lamprey genome encodes one or two SECIS binding proteins and therefore whether or not SBP2/SBP2L divergence occurred before or after the radiation of jawed vertebrates. Since we have only been able to identify two SBPs in vertebrates, this would suggest, in conjunction with the 2R hypothesis, that additional copies were lost soon after genome duplication.

**Figure 6:**
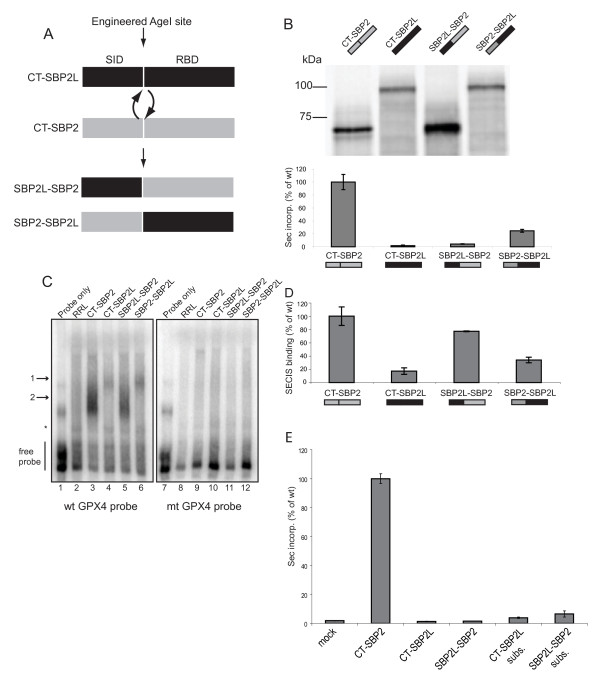
**The SID-like region in SBP2L does not promote Sec incorporation**. (A) Schematic of the SBP2L/SBP2 domain swap. (B) 2 fmol of the indicated [^35^S]-Met labeled *in vitro *translated proteins (top gel) were added to an *in vitro *translation reaction containing a luciferase Sec incorporation reporter. Luciferase activity (Sec incorporation) is expressed as a percent of that obtained with wild-type CT-SBP2 (bottom graph). (C) 8 fmol of [^35^S]-Met labeled *in vitro *translated proteins were incubated with [^32^P]-labeled wild-type (wt) or mutant (mt) GPX4 SECIS elements and resolved on a 4% non-denaturing polyacrylamide gel. Arrow 1 marks CT-SBP2L and SBP2-SBP2L complexes, arrow 2 marks CT-SPB2 and SBP2L-SBP2 complexes. The asterisk marks a probe shift resulting from an unidentified component in rabbit reticulocyte lysate. (D) Graphical representation of EMSA data shown in (D) expressed as the percent of probe shifted relative to that obtained with wild-type CT-SBP2. (E) 2.4 fmol of the indicated [^35^S]-Met labeled *in vitro *translated proteins were added to an *in vitro *translation reaction containing a luciferase Sec incorporation reporter. Mock contains no added *in *vitrotranslated proteins. 'CT-SBP2L subs.' and 'SBP2L-SBP2 subs.' indicate proteins bearing the following substitutions: D494G, SKA556PLM, EK563QR and A567P (human SBP2L numbering). Sec incorporation is expressed as a percentage of CT-SBP2. All data are mean ± SEM of three independent experiments.

While mammalian SBP2L has not been fully biochemically characterized, it is known that human CT-SBP2L (aa 467-1101), which contains the entirety of the SID and RBD, is not able to support Sec incorporation and weakly binds the GPX4 SECIS element compared to rat CT-SBP2 (aa 399-846; Figure 6) [20]. Nonetheless, the presence of just SBP2L in invertebrate deuterstomes and conservation of the SID and RBD between vertebrate SBP2 and SBP2L suggests that vertebrate SBP2L may play a role in regulating selenoprotein expression via interactions with SECIS elements. This begs the question as to whether there is a correlation between SECIS structure and the occurrence of SBP2L as the only SBP in an organism. To examine this potential correlation, we turned to the selenoprotein rich sea urchin *S. purpuratus*. We identified 14 SECIS elements from *S. purpuratus *using the SECISearch algorithm in combination with the selenoprotein identification procedure previously described [21]. These were compared them to all 26 human SECIS elements in a multiple sequence alignment. Interestingly, all 14 *S. purpuratus *SECIS elements were of the Form 2 class. Aligning the *S. purpuratus *SECIS elements failed to identify any novel characteristics that might explain the preferred association with SBP2L. Additionally, aligning human SECIS elements with those from *S. purpuratus *and constructing a phylogeny with PhyML did not generate any clades that could suggest potential human SBP2L targets (data not shown). Recently, Takeuchi et al [12] reported that SBP2 from *D. melanogaster *is only able to bind Form 2 SECIS elements but that Form 1 binding is conferred when the SVRVY^95-99 ^sequence in the SID was changed to that found in human SBP2 (IILKE^535-539^). However, the existence of only form 2 SECIS elements in *S. purpuratus *was not likely constrained by SBP2L sequence in this region as it contains a VILKE motif. These results suggest that determinants in the SECIS element for SBP2L interaction lie outside of the sequences tested or in structural elements that cannot be detected by sequence alignments. Overall, these results suggest that SBP2L is a *bona fide *Sec incorporation factor in invertebrate deutoerstomes, but that it may have diverfed to fulfill a separate function in vertebrates.

### Conservation between SBP2 and SBP2L is not sufficient to promote Sec insertion

Since SBP2L possesses an RBD that differs from that in SBP2 at positions known to be required for SECIS binding, we hypothesized that a fusion protein consisting of the SBP2L SID and the SBP2 RBD may be able to support Sec incorporation *in vitro*. To test this, we engineered an Age I site in the non-conserved regions between the SID and RBD of CT-SBP2L and CT-SBP2 and swapped the domains yielding the following chimeric proteins: SBP2L^467-647^/SBP2^586-846 ^(SBP2L-SBP2) and SBP2^399-585^/SBP2L^648-1101 ^(SBP2-SBP2L). Figure 6A shows a schematic of the domain swap. Sec incorporation activity of the chimeric proteins was determined by adding 2 fmol of each protein to an *in vitro *Sec incorporation assay that measures the translation of luciferase mRNA containing an in-frame UGA codon and GPX4 SECIS element in the 3'-UTR [22]. Figure 6B shows the amount of Sec incorporation activity obtained with each protein relative to that obtained with CT-SBP2. As expected, CT-SBP2L was unable to promote Sec incorporation. Surprisingly, the SBP2L-SBP2 chimera was also unable to promote Sec incorporation while the SBP2-SBP2L chimera retained 20% Sec incorporation activity compared to CT-SBP2. These data indicate that despite strong similarity between the SID regions in SBP2 and SBP2L, the latter is unable to promote Sec incorporation in this assay even when appended to the SBP2 RBD. In order to determine whether the lack of Sec incorporation activity observed for the chimeric proteins was due to a lack of SECIS element binding, we tested whether or not they could bind the GPX4 SECIS element by electrophoretic mobility shift assay (EMSA). *In vitro *translated proteins (8 fmol) were incubated with wild-type or mutant [^32^P] UTP-labeled SECIS elements and the complexes resolved by non-denaturing gel electrophoresis. This amount of CT-SBP2 is within the linear range of this binding assay (data not shown). As expected, CT-SBP2 shifted wild-type but not mutant SECIS elements in which the AUGA motif in the SECIS core was deleted (Figure 6C, compare lanes 3 and 9). Consistent with previous results, CT-SBP2L SECIS binding activity was only ~20% relative to CT-SBP2 (Figure 6C, compare lanes 3 and 4; Figure 6D). Interestingly, the SBP2L-SBP2 chimera specifically bound the wild-type SECIS element at ~80% the level of CT-SBP2, while the SBP2-SBP2L chimera provided only 40% binding relative to CT-SBP2 (Figure 6D). These results show that a lack of SECIS element binding does not explain the inability of the SBP2L-SBP2 chimera to support Sec incorporation. In addition to SECIS element affinity, the SID is thought to play a role that is likely downstream of SECIS binding, such as promoting an eEFSec or ribosomal conformational change. The key residues found to be required for a function downstream of SECIS element binding were from P513 to P524 [2].

In light of this we wanted to know whether divergence of SBP2L and SBP2 in these regions were responsible for the inability of the SBP2L-SBP2 chimera to promote Sec incorporation. Specifically, a series of mutations were made such that either CT-SBP2L or the SBP2L-SBP2 chimera contained the following substitutions: D494G, SKA556PLM, EK563QR and A567P (human SBP2L numbering). Using Figure 3B as a reference, these substitutions cover the sites annotated as G454 and Q520 (human SBP2 numbering). CT-SBP2 and the wild-type or mutant versions of CT-SBP2L and the SBP2L-SBP2 chimera were analyzed for their ability to support Sec incorporation *in vitro*. Figure 6E shows that none of the substitutions resulted in an active form of either CT-SBP2L or the SBP2L-SBP2 chimera. These results demonstrate that the chimeric protein either contains a negative element that actively prevents Sec incorporation or that it is missing an as-yet undetermined element that is required. Further analysis of the physical interaction between the SBP2L SID and the SBP2 RBD will be required to determine which is the more likely explanation for the inability of the SBP2L SID to support Sec incorporation even when SECIS binding is restored.

### The SBP2 L7Ae RNA binding domain arose from ribosomal protein L30

**Figure 7:**
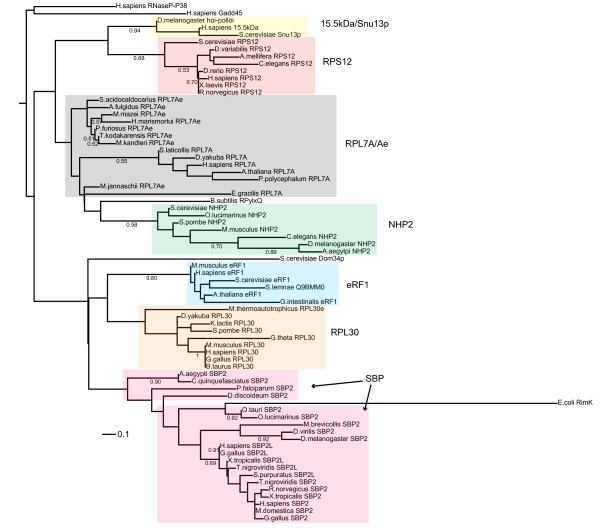
**Phylogenetic tree of L7Ae core motifs**. A phylogenetic tree of core motifs from L7Ae family members was inferred by maximum likelihood methods using PhyML with 500 bootstrap replicates. The tree was re-rooted with the Gadd45/RNaseP-P38 clade as the outgroup. Bootstrap values less than 0.5 are not shown. Scale bar represents amino acid substitutions per site.

Having established the phylogenetic relationship of metazoan SBPs, we sought to determine whether we could elucidate the origins of SBP2. As mentioned above, SBPs contain an L7Ae RNA binding motif. This motif was identified by Koonin and colleagues [23] as a 32 amino acid motif (L7Ae core) in primarily ribosomal and ribosome-associated proteins such as ribosomal protein L30 (RPL30), RPS12, the translation termination factor eRF1, and RPL7A, among others. This motif features a universally conserved glycine residue 15 amino acids upstream of a 3 residue hydrophobic cluster [23]. The SBP2 L7Ae motif is well characterized [4,13,24] and has been shown to be responsible for sequence-specific SECIS element binding, although unlike RPL30 [25], for example, high affinity binding requires a large stretch of sequence N-terminal to the core motif. In order to determine a putative evolutionary origin of the L7Ae motif in SBP2 and SBP2L we aligned them with L7Ae core sequences from other L7Ae-containing proteins using MUSCLE (Additional file 4) and generated a maximum likelihood tree in PhyML using the WAG substitution model. The tree suggests that the SBP clade shared a common ancestor with the RPL30 and eRF1 clades (Figure 7). Interestingly, both RPL30 and RPL7A have been shown to possess SECIS binding activity, and the former was able to stimulate Sec incorporation activity in cells while the latter was inhibitory. Whether the ability of these proteins to bind SECIS elements is functionally significant, however, remains to be determined.

### The SBP2 SID is of uncertain origin

While the SBP2 RNA binding domain has clear evolutionary roots in ribosomal proteins, the SID is a protein domain that is unique to the SBP family with no apparent homology to other protein domains as efforts to identify molecular relatives by PSI-BLAST failed. Thus, it is possible that the SID arose *de novo*. Regardless of its origin, the conservation of the SID core motif in the eukaryotic taxa presented here (K517-R543) suggests that it arose once in eukaryotic evolution and that organisms such as *D. discoideum *and *Plasmodium vivax *with poorly conserved SID sequences likely diverged to adapt to as-yet unidentified unique requirements (Figure 5A).

### Archaeal Insight into SBP2

**Figure 8:**
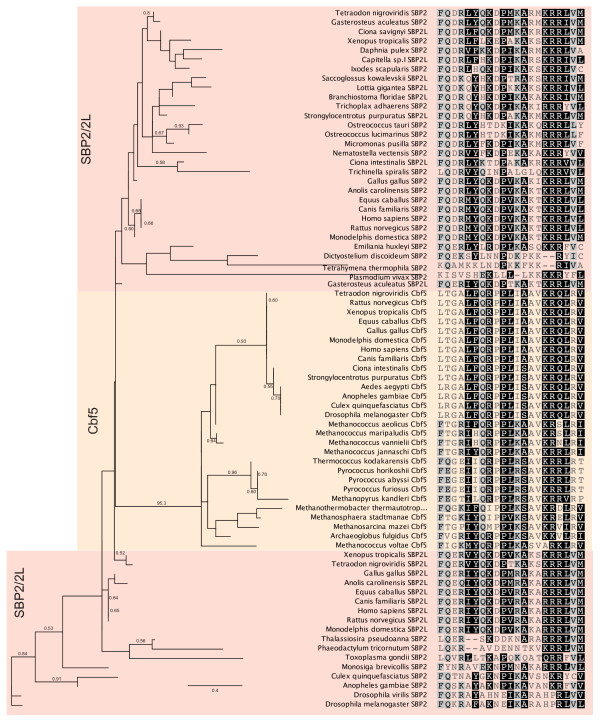
**Convergence of a short motif in Cbf5 and SECIS binding proteins**. Maximum likelihood tree of Cbf5 and SECIS binding proteins inferred using PhyML with 1000 bootstraps. Bootstrap values below 0.5 are not show. The alignment used to generate the tree is shown to the right.

Caban and colleagues recently performed structure/function studies on the SBP2 RNA binding domain including its L7Ae motif [13]. In this work they noted that SBP2 residues in the region of RFQDR^654-658 ^N-terminal to the L7Ae core were required for Sec incorporation but not SECIS element or ribosome binding. Interestingly, these residues are not conserved among the various subgroups of the L7Ae superfamily. Accordingly, we sought to determine whether or not this motif and residues in its vicinity had a discernible relative. A BLASTp search against the Protein Data Bank with relaxed settings and rat SBP2 as a query expectedly yielded hits of various L7Ae family members. However, this search also produced a hit for *Methanococcus jannaschii *Cbf5, the H/ACA sRNA guided pseudouridylate synthase [[26], reviewed in [27]]. Querying the CDD with rat SBP2 and relaxed settings (Expect Threshold = 10) also yielded a hit against Cbf5 (Expect = 2.3). This corresponded to rat SBP2^642-666 ^and and *Pyrococcus horikoshii *Cbf5^125-150^. In addition, PSI-BLAST using full length rat SBP2 as the initial query independently led to the identification of Cbf5 as a potential relative (data not shown). Notably, the only region of significant similarity is that between RFQDR^654-658 ^and the universally conserved Gly residue in the L7Ae RNA binding motif (G676). Figure 8 shows a multiple sequence alignment and phylogenetic tree illustrating the relationship between the Cbf5 and SBP2. The conservation of this region among the Cbf5 group from archaea to mammals is quite high with strong clade support, but the highest level of conservation is between archaeal Cbf5 and vertebrate SBP2. The substantial divergence between the Cbf5 motif and that observed in SBP2 from unicellular eukaryotes and protostomes and the short length of the motif suggests that it may have arisen as a result of convergent evolution.

This finding is intriguing as Cbf5 is known to form a complex with L7Ae as well as Nop10, Gar1, and box H/ACA s(no)RNAs, which contain kink-turn elements similar to that in SECIS elements [1,27]. *In vivo*, Cbf5 catalyzes the site-specific pseudouridylation of rRNA with the aid of guide H/ACA s(no)RNAs. Minimally, Cbf5 and Nop10 are required for guided pseudouridylation but maximum activity requires all four H/ACA sRNP proteins [28-31]. The crystal structure of an archaeal H/ACA sRNP, showed that the Cbf5 region found in our BLAST searches is physically distant and makes no direct contacts with L7Ae [28]. Part of the Cbf5 region from our BLAST searches, though, interacts with the accessory protein Gar1, which has structural homology to domain II of EF-Tu, the bacterial translation elongation factor [28,32], suggesting that this region within SBP2 may be required for eEFSec binding. Additionally, part of the Cbf5 BLAST hit forms a 'thumb loop' (Cbf5 β7-β10 loop) that has been proposed to stabilize substrate RNA [28]. From this, we speculatively propose a model in which this Cbf5-like region within SBP2 serves as a switch that regulates eEFSec/Sec-tRNA^Sec ^interactions.

### Mosquito SBP2: Science imitates Nature

**Figure 9:**
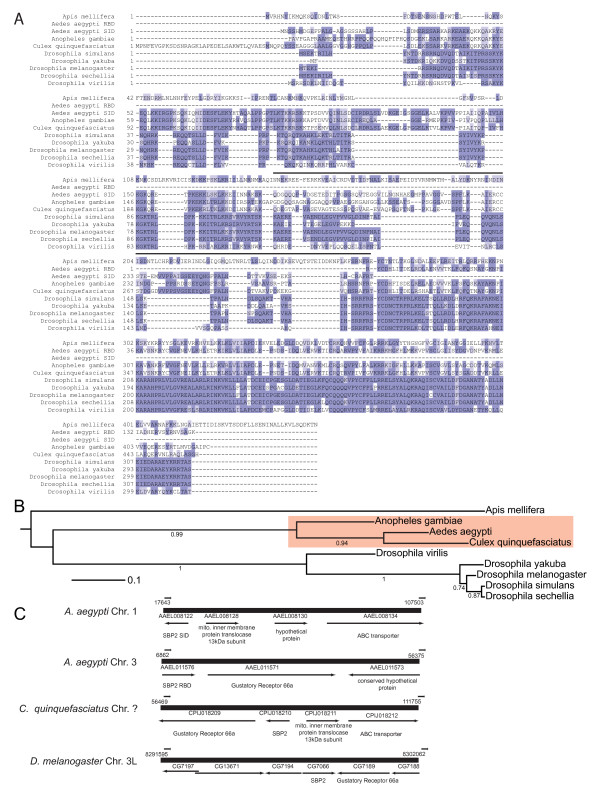
***Aedes aegypti *SBP2 is coded by two genes**. (A) Multiple sequence alignment of insect SBP2 from the indicated species. Residues were colored using JalView based on BLOSUM62 score. The black line above the alignment denotes EST coverage of residues in the predicted *A. aegypti *SID peptide that are in an EST corresponding to the *A. aegypti *RBD. (B) Maximum likelihood tree of the insect SBP2 sequences used in panel A. In order to build the tree, the *A. aegypti *SID and RBD sequences were combined into one sequence file. (C) The genomic contexts of SBP2 from *A. aegypti *and *C. quinquefasciatus *and *D. melanogaster *were ascertained from Entrez Gene at NCBI. Arrows indicate gene orientation and gene names are as indicated.

Chapple and Guigó recently investigated evolutionary relationships of insect selenoproteins and Sec incorporation factors [7]. In that work, a multiple sequence alignment of insect SBP2 showed that *Aedes aegypti *SBP2 contains an RNA binding domain but apparently lacks a Sec incorporation domain. This is striking as SBP2 from other insects, including the mosquitoes *A. gambiae *and *Culex quinquefasciatus*, contain a Sec incorporation domain [7]. We therefore performed a BLASTp search with default parameters but limited to *A. aegypti *(taxid: 7159) using *C. quinquefasciatus *SBP2 as a query. This search yielded the previously identified *A. aegypti *SBP2 RBD [7] and *A. aegypti *hypothetical protein AaeL_AAEL008122 (Expect = 7e-85; hereafter *A. aegypti *SID) which has 66% identity and 76% similarity to *C. quinquefasciatus *SBP2^3-275^, suggesting that the SID is encoded by a separate gene in *A. aegypti*. A BLASTp search against *A. aegypti *using the SID of rat SBP2 (aa 504-530) as a query also identified the *A. aegypti *SID (Expect = 1e-04). A multiple sequence alignment of representative insect SID and RBD sequences is shown in Figure 9A. Phylogenetic inference of insect SBP2 using the WAG substitution model in PhyML placed *A. aegypti *SBP2 with that of other mosquitoes with strong bootstrap support (Figure 9B).

BLASTx analysis of 6.86 kb of genomic DNA [Refseq: NW_001811341.1] upstream of the *A. aegypti *SBP2 RBD coding region did not detect any sequences similar to SBP2. Consulting the Entrez Gene database at NCBI [33] for these genes showed that the *A. aegypti *SID and RBD were found on chromosomes 1 and 3, respectively, further suggesting that SBP2 in *A. aegypti *is comprised of two separately encoded proteins. Additional evidence that *A. aegypti *SBP2 is split comes from examining its genomic context relative to that of other insects. Specifically, *D. melanogaster *and *C. quinquefasciatus *SBP2 are linked to the locus for gustatory receptor 66a, which flanks the *A. aegypti *SBP2 RBD (Figure 9C). Additionally, the SBP2 locus in *C. quinquefasciatus *is linked with a mitochondrial inner membrane protein translocase and an ABC transporter, both of which are also linked with the locus for the SBP2 SID in *A. aegypti *(see genomic diagram in Figure 9C). Thus the *A. aegypti *SBP2 SID and RBD were likely separated by a translocation event. While the genomic sequences and predicted peptides suggest the *A. aegypti *SID and RBD are separate proteins, an EST search revealed that 2 ESTs (Genbank: DV248278.1; DV248276.1) corresponding to the RBD also encode amino acids 178-275 of the predicted SID peptide. It is also noteworthy that these ESTs do not code for a methionine residue upstream of the RBD suggesting that their 5' ends are incomplete and that the complete mRNA could code for the entirety of SBP2. This would be possible if the SID and RBD transcripts were joined by *trans*-splicing which has been previously reported in *A. aegypti *[34]. In the absence of an EST set that covers the entirety of *A. aegypti *SBP2 it could still be possible for the SID and RBD to function as separate proteins as supported by recent work showing that combining recombinant rat SBP2 SID and RBD that have been physically separated can promote wild-type levels of Sec incorporation *in vitro *[2].

## Conclusion

This report represents the first phylogenetic analysis of SBPs across eukaryotic taxa. Since vertebrates possess two SBPs, we annotated determinants that serve as identifying motifs in SBP2L and SBP2. We showed that SBP2L is the likely progenitor of mammalian SBP2 and as the only form of SECIS binding protein in invertebrate deuterostomes, SBP2L is likely required for Sec incorporation in these organisms. However, the role of SBP2L in vertebrates will require further experiments to elucidate its function, and we expect forthcoming experimental evidence to support this hypothesis. Additionally the retention and loss of LSAD and PVFQ motifs in several taxa suggest that SBPs are subject to diverse evolutionary pressures. Conservation of the SID and RBD in unicellular eukaryotes and metazoans suggests that SBP2 arose once in eukaryotic evolution.

The origins of the SID and RBD, the essential domains for SBP2 function, may provide insight into the mechanism of Sec incorporation. The apparent phylogenetic relationship between the SBP L7Ae motif and RPL30 could provide insight into SBP2-ribosome interactions or reiterate a possible role for RPL30 in Sec incorporation as suggested by Chavatte [25]. The origin of the SID, however, is unclear and its relation to other proteins may require structural analysis. Analysis of insect SBP2, specifically that of *A. aegypti*, showed that its SID and RBD are coded by separate genes. Future use of *A. aegypti *cell culture could provide a unique system in which to study Sec incorporation. Based on the taxa sampled here, we propose that SECIS binding proteins arose once in eukaryotic evolution and diverged substantially as in the case of *P. vivax*, *T. gondii*, and D. *discoideum*. Additionally, the presence of SBP2L in some protostomes (*C. sp. I and L. gigantia*) but not in others (i.e. insects) suggests that SBP2L sequences occurred in the last common ancestor of protostomes and deuterostomes followed by divergence in different lineages. Lastly, a gene duplication event, possibly as the result of whole genome duplication, generated the paralogous pair of SBP2 and SBP2L in vertebrates.

## Methods

### BLAST and Identification of SBP2 homologues

BLAST searches were carried out as described in the text. Annotation of SBP2 from organisms with sequenced genomes was performed in the manner outlined in Additional file 2. The presence of SBP2L or SBP2 was determined by BLAT (UCSC Genome browser [35,36]) or BLAST (Joint Genome Institute organism-specific webservers and genomic DNA was used as input for *ab initio *gene prediction using GenomeScan or the FGenesH webserver [8,9]. Predictions were refined based on EST data from the organism of interest or related taxa when available.

### Constructs

CT-SBP2 and CT-SBP2L constructs were created by TOPO-TA cloning the coding regions for rat SBP2^399-846 ^and human SBP2L^467-1101 ^into pCR3.1 (Invitrogen). SBP2/SBP2L chimeric constructs were made by introducing silent mutations in the coding regions rat for SBP^399-846 ^(CT-SBP2) and human SBP2L^467-1101 ^(CT-SBP2L) in pCR3.1 to create Age I restriction sites. These constructs were then double digested with BstX I/Age I or Age I/Xho I. The SBP2L-SBP2 chimera was generated by ligating the 555 bp SBP2L BstXI/AgeI product and the 838 bp SBP2 Age I/Xho I product into BstX I/Xho I digested pCR3.1. The SBP2-SBP2L chimera was generated by ligating the 576 bp SBP2 BstX I/Age I product and the 1.4 kb SBP2L Age I/Xho I product in BstX I/Xho I digested pCR3.1. Substitutions in CT-SBP2L and SBP2L-SBP2 were generated by site-directed mutagenesis. Sequences of all constructs were verified by automated DNA sequencing.

### *In Vitro *translation

Plasmids encoding wild-type or mutant CT-SBP2, CT-SBP2L, and chimeras in pCR3.1 were linearized with Xho I and transcribed with T7 RNA polymerase using the mMessage kit (Applied Biosystems). mRNAs were translated in nuclease treated rabbit reticulocyte lysate (Promega) in the presence of [^35^S]-Met as directed by the manufacturer. Protein quantitation was performed by PhosphorImager analysis as previously described [13].

### Sec incorporation assay

Sec incorporation assays were performed by adding 2 fmol (Figure 6B) or 2.4 fmol (Figure 6E) of *in vitro *translated protein, as described [2], to an *in vitro *translation reaction containing 50 ng of a luciferase reporter bearing a Cys258Sec mutation and a GPX4 SECIS element [22].

### Electrophoretic mobility shift assays (EMSA)

[^32^P]-labeled wild-type and mutant GPX4 SECIS elements were transcribed as described by Caban [2007]. SECIS binding of *in vitro *translated proteins was assayed by incubating 8 fmol with 20 fmol of wild-type or mutant (ΔAUGA) SECIS probes in 1 × PBS supplemented with 250 μg/mL yeast tRNA (Sigma), 10 mM DTT, and 5 μg soybean trypsin inhibitor (Sigma). Final reaction volumes were 20 μL. EMSA reactions were incubated for 30 min at 37°, resolved on 4% non-denaturing polyacrylamide gels, and visualized by PhosphorImaging. Percent shifted probe was determined with ImageQuant software by subtracting background from the RRL only lane and normalizing to CT-SBP2.

## Abbreviations

aa: Amino acid; Sec: Selenocysteine; SECIS: Sec Insertion Sequence; SBP: SECIS binding protein; SBP2L: SBP2-like protein; SID: Sec incorporation domain; RBD: RNA binding domain; RP: Ribosomal protein; EMSA: Electrophoretic mobility shift assay; RRL: Rabbit reticulocyte lysate; UTR: Untranslated region; EST: Expressed Sequence Tag;

## Authors' contributions

JD performed all wet-lab experiments, sequence alignments, gene predictions and phylogenetic analysis, contributed to the writing of the manuscript and preparation of figures and PRC initiated the project, performed sequence alignments and phylogenetic analyses and contributed to the writing of the manuscript and preparation of figures. Both authors read and approved the final version.

## Supplementary Material

Additional File 1 Multiple sequence alignment of all SBP2 and SBP2L sequences used for Figures 2 and 3. Click here for file

Additional File 2 A generalized workflow for identification of SBP2 or SBP2L in organisms for which these proteins were not annotated. Click here for file

Additional File 3 **List of SBP2/2L sequences used in this paper**. Accession numbers are provided for sequences available at NCBI and predicted sequences are provided in full. Underlined residues indicate regions supported by ESTs. NCBI annotated sequences marked with an asterisk were refined based on EST and/or genomic DNA data (italicized residues). Note that some predicted sequences are likely to be partial due to lack of EST coverage or inability of gene prediction programs to correctly identify a start codon. Click here for file

Additional File 4 Alignment of the core L7Ae motifs used to generate the phylogenetic tree in Figure 7. Click here for file
