# Supplementary figures and images for: Evolutionary history of selenocysteine incorporation from the perspective of SECIS binding proteins
Source: BMC Evol Biol. 2009 Sep 10;9:229. doi: 10.1186/1471-2148-9-229 (PMC2746813; doi:10.1186/1471-2148-9-229)

# Workflow for annotating SBP2 and SBP2L and refining incomplete SBP2 annotations

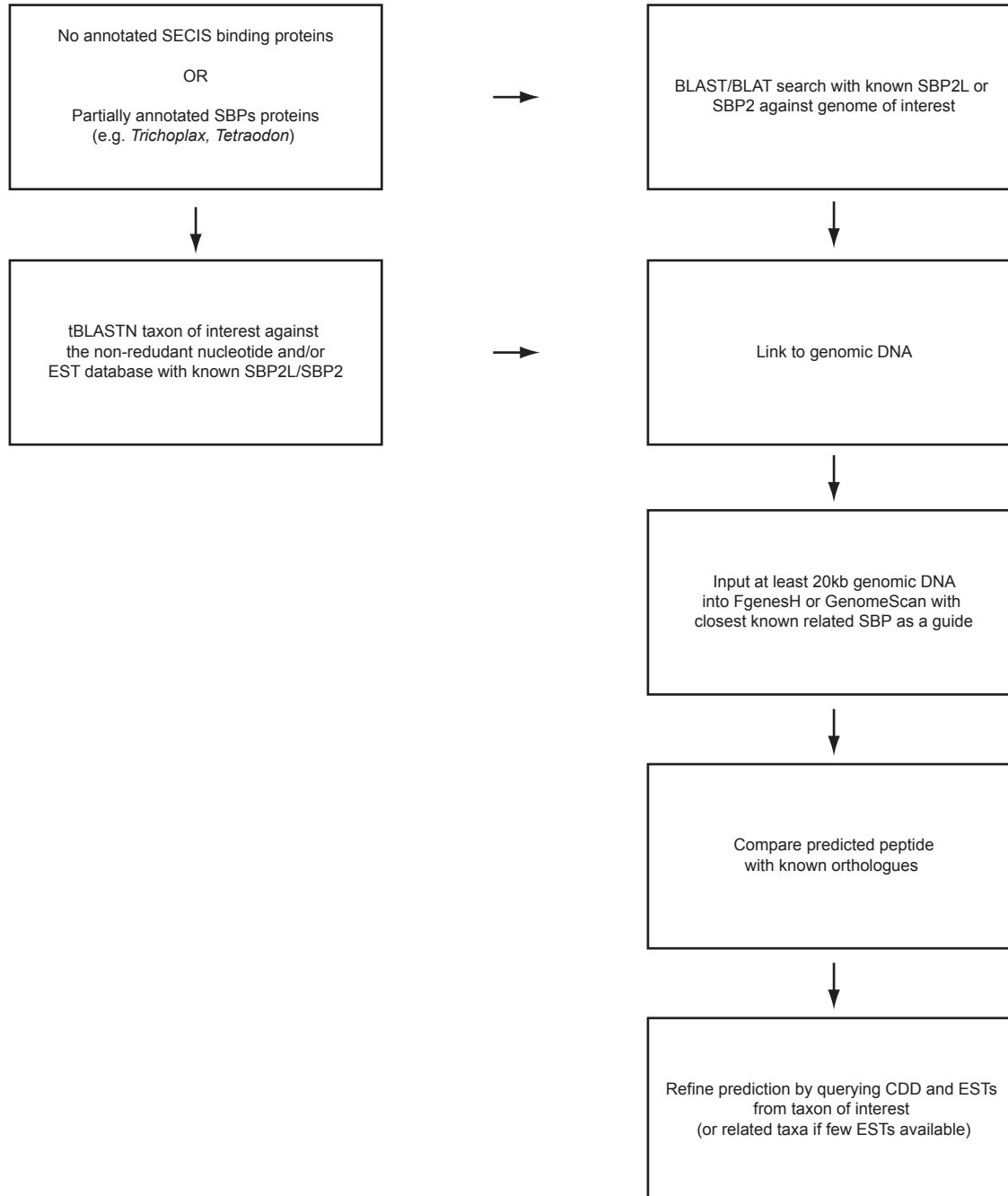

Supplement: Additional File 2 — A generalized workflow for identification of SBP2 or SBP2L in organisms for which these proteins were not annotated. [file 1471-2148-9-229-S2.pdf]
