# Supplementary material for: Evolutionary history of selenocysteine incorporation from the perspective of SECIS binding proteins
Source: BMC Evol Biol. 2009 Sep 10;9:229. doi: 10.1186/1471-2148-9-229 (PMC2746813; doi:10.1186/1471-2148-9-229)
Supplement: Additional File 3 — List of SBP2/2L sequences used in this paper. Accession numbers are provided for sequences available at NCBI and predicted sequences are provided in full. Underlined residues indicate regions supported by ESTs. NCBI annotated sequences marked with an asterisk were refined based on EST and/or genomic DNA data (italicized residues). Note that some predicted sequences are likely to be partial due to lack of EST coverage or inability of gene prediction programs to correctly identify a start codon. [file 1471-2148-9-229-S3.pdf]

Predicted Sequences

Aedes aegypti SID (Refseq: XP_001658947.1)

Aedes aegypti RBD (Refseq: XP_001661740.1)

Anolis carolinensis SBP2

MASERETSQGIKLSAEVKPFVPKYAAVTVAWSEPSEACVFPRYYTTCYPYVQDPSVDKHQVYTQ
DLPWDSTSTLPHYVSQNVMGCTSLGEYTSSPYTPDAAPNVYTVPNFRSENNNSKLFSSSRQGRD
YAKQTPVSRKEAAYHPRRRPRDFEPKLESKRPDGSDSPNRSTESSSYTALHGRNPIKSEPAFEV
NLSDFPKLQSTRSCNLLNFQKQTWGPLGSAEGDKMPRKPPEKAVSALVTKEGEECLKRNLPTRP
FTSPPDPGVSQVHLDYFTETPKSCATVLSQTPKRTVSTPAASPSTNHYENKGKQGVLEDRKSEP
KGNASDSEPEEIVEKKKKKKRNKKKAKASSETENVPNKSIIYQEPPRIEVQIFIFLVISRFLCS
LPGITGQFGVCRNSGITMAYNETSKSGGKKSHLPVQLDLGNMLAALEQKQQAEKTKHSPKPVVL
SVGGTVTLPSKEPMNQTKNQQLNQGNSPHNPLDSSSPLIKKGKQREVPKAKKPTSLKKIILRER
EQRKQQHLLEQMATPKDSDAAQDAESTAENQGYSEDNKAESLEDTSIDPDILAQDGPEVDLKCT
ELNGSYTESVLDPVQLKSNLPKIHSRRFRDYCTQVLSKEVDSCVTDLLKELVRFQDRLYQKDPV
KAKTKRRLVMGLREVLKHLKLKKLKCVIISPNCEKIQSKGGLDETLHLIIDSACEQNIPFVFAL
NRKALGRCLNKAVPVSVVGIFSYDGAQDYFHKMVELTMEARQAYKDMISALERESAEEESGSDC
TPSKNPYSESRPLDTVEVPPPESEEDVPDYKIWKQKLEEEYKPYMLELEKQLTAEMLPMGFEEP
Q

Anolis carolinensis SBP2L

NVKLSAEVEPFIPQKKGPDSLMMPMALPNENGGVNGVEAAPIPSYLITCYPFVQENQSNRQLPL
YNNDIRWQQPNPNPAGPYLAYPILSAQPPVSTEYTYYQLMPAPCAQVMGFYHPFPAPYSAPFQT
ASAMNTVTTECTDRPTQPNQVFPMCSQRSRSASRGSIVQKQQQPLQTHIKSKRPPVKSVATQKE
TCASGPETRPKIVLLVDACQQTDFPSEIASKSLSESMSTMHWKPKTRRRRSSHPAESSSEQGAS
EADIDSDSGYCSPKHSNQAAAVTSRSVESAAGNILEPSVHPAGATWANVSSQATQKKPWMERTP
AFSRGGRQAEQHNSSQDEEEFPELNSDSGNSKGGNMQLKNAPKVLDDLPENSPINIVQTPIPIT
TSVPKRAKSQKKKALAAALATAQEYSEISMEQRKLQEAVSKAAGKKSKTPVQLDLGDMLAALEK
QQQAMKARQITNTRPLSYTVGNAAPFHTKESTNRKSLAKGQPSVGCLNPLDSTSPKVKRGKERE
LSKLKRPTALKKIILKEREEKKGRLSVDHTIVGADEEQEVDLNLTPNQSEELASQEEPGLSVPS
DTSLSPASQNSPYCMTPVSQGSPASSGIGSPMASSTITKIHSKRFREYCNQVLSKEIDECVTLL
LQELVSFQEQIYQKDPMRAKAKRRLVMGLREVTKHMKLSKIKCVIISPNCEKIQSKGGLDEALY
NVIAMAREQEIPFVFALGRKALGRCVNKLVPVSVVGIFNYSGAENLFNKLVSLTEEARKAYRDM
VAAMEQEQEEALKNVKKVPHHMGHSRNPSAASAISFCSVISEPISEVNEKEYETNWRSMVETSD
GLEASENEREASFKSAVPEKPGNSNKVIVNKELSLAAVGVVTVSAQVKAPSGKEEVKPDDNLEW
ASQQSTDTGSLDGSCRDILNSSMTSTTSTLVPGMLEEEDDEEDEDEDDYTHEPISEEVQLNSRI
ESWVSETQRTMETLQLGKSLSGGEDDNAEQSEDEELETPEQVESVVESEEWTAEKHSNKAQQKP
AVSTSLDTTPMDSNYIP

Anopheles gambiae SBP2

PAVPGAPRAAMEQETHNRPPQQPQQHQPIPHQSDGEKLSARKARKEAERQKKQAKKYEEQLKKI
RGPKSQKLQIIDESFLEKYRHVQSLPPGPTLKTKKRSKKAPTDVVQINLSECIREQLAGGERME
PKPIVPIVPQQPLLLLHKGKQREVPKEKKLTRLKKDIIRSRTEKGAPGDGQQSAGNAGGAQQPV
PAEGGKGANGGLLKSSEATSPSGGGAVDSPFLKAIERCCINDGPPPSRDSSEYQNGFPSLRVSA
AVSRPTALRHSRNFRPYCDHFISDELRELAEDVVVKLFSFQSKAYAKNPIKAVANKRFVVGFNE
VLKLLELRKVRLVLIAPDLEPNETIDQMVANVKMLCRQSQVPYLFALKRRKIGFHLLKKAPISC
VGVLSYAGCDDTVKQMLSVVEQERESYRTLMVDGAIPC

Apis mellifera SBP2 (Refseq: XP_001122154.1)

Canis familiaris SBP2 (Refseq: XP_533552.2)

Canis familiaris SBP2L (Refseq: XP_544672.2)

Capitella sp.I SBP2L

MFKLSAEAKSFVPRHATPADESGVLPYYVTNCYPFVQTVEPSNNAQVLSRDAGVRAAWNSCFSP
RESEVASLHGPPRNSRPKNSRGLLGVHPMSVHAAPYTALPFPTPRGFSPGGGAIGGMYPRTPPR
WNTDPPPPLGVVPPQRSIWWRGPRPAFASNGVNLKHHPANQPTARKRSKSKTIICMDAAFQTGL
LCIVSAIAHLFCLDFSEEIARYTLAERPNSLYCKSCHVSTDSEEVDSDSGYSSPLHRKNQISNG
TDASPGSLLQSKQRALGAAVSGAPSVMSYSSVVSRAASAKPTQCQRSESGIKTDNSEAEGDPGS
GRKKQRGKRKKKRRKSAGDEEVPPTEQKPPEQEEHAEEELPELNFQDEEEFPNLLSGGVPPPAA
PLSYSEVLRHSYSASGQSQESSLSHSTTGGQRSADRSHASTPNSFDSKGGKESKSARKRRKRRE
QANTAADNELAEISLEQEVLRELSIVHNMDPKAAAIKQQEKSSSRLPPAPVAKQPPSTGKKSKQ
PIALNIADMIDALEKAQDVPAKKPLKSEKAAPGGAREGVRVANALDASAPVKRGKEREAPRNKK
PSPLKKVILKEREEKKRLRLLEETGPISPSETSASASQIQSGLPLDSDLSQDGVSSKASSMDCG
TTTPVTVDMSPISQTSPVSMSPLSGSPFASGINSPIACGLAGLGIVPKALDDIDASVLMKIHSR
RYREYCKQVLNKDIDSCCTALLQDLIRFQDRLFHKDPIKARSKRRIVLGLREVTKHLKLKKLRC
VIISPNLERIQSKGGLDEALNNILQICQEQTVPFVFALGRRALGRACAKLVPVSVVGIFNYEGS
EGNFKKLMDLMGRAREAYDQMVSNIQKELQEHRSSTRTVASSVGTQLFPHMGHSRTPSACSAIS
FTSSILSEPISENYPRSEPETDSRGYEIVKKEEEEEEEEEEEEEEEEEDQSSVIDKIATLTEID
DGHEADTEEDHYKVKAADLPPIDSIHSATLDLSSEILSQHSSKTLELAKSESKSCKVAFAEDVT
SLGGVSGKQAPTSSSVNKDRIELWVADTTKQIEHLGESKQ

Ciona intestinalis SBP2L (Refseq: XP_002123233.1)

Ciona savignyi SBP2L

MKLRTNLRAEVPPFVPRREWTEESMDPQSGRPLPRYVTTCYPFVQDNQDERRMQHGANFNQRFP
TPALRNGTTYANVGYLDSSSNSAAFTNVNQFAMGTVSQNFSGLPFEGNLADTAHITAVYGDSIQ
CQYSPNPSSVVVHKSSSLKTRSRGSSGKRILKRNVGTQKEANRGSPSPELVDNCQQTDFPISIA
SKSLTDHPSLLRKPGRSRRRRETLVYTSGAYDSSSDHADVDSDSGYYSPKHRLVHAKSGGTSTH
GLNARNEKDSVPQVIYITPSNVSAHGPMFQVHPSSATNHLFHNSLSPHVTPPPTSLLGYGQPGP
PIIFSPPPNPMLANRPSPGFPLHPSLIQTSNGNQPIGVALTCGEALFVKQVFLYILCEFRAVEK
KRRALVTFSQQAFMLRVHQSMNPAGPAERTIKKKAARLASSGSSTSNWNTNMAFHAGYWSNGPN
PFHHQQNFRNPSLDPRSQRKFNKFDNRSKSPSYGGHTNHHQMGKSSTMERQLRDDSPIVLNSHN

NPSINGLHLPEDNTLFMLSDNQEFPGLGGNLYTTSTSAPGNVFSYSAAVMGKIPRSSQSNVQNQ
VYPPSNSNEKAQAQKSKKRRKKAERAAKAADEEYAEISKEHENIQKVFKKTASGRNKSKTPVLD
FSEVMTKLEEKKIIDSSQKGGASEKLKDNAAAKKPPLNIRQTAVSMITPMNALDGTAPMIKKGK
EREVPKVKKPSALKKVILKEREEKKEHHLKQLTTMPVPAPDVPPYPASIYRAINTTDESKGESV
AAPDSVDIIPATVPQIHSRRYREYCCQVLDKRIDETCTMMLQKLVQFQDRLFQKDPMKAKRKRR
LVMGLREVTKHLKMKKLRCIFISPNLEKIQAKGGLDDALQQILNLCEEQNVTYVFALGRKALGR
AVSKMVPVSIVGVFDYSGAEEQYKQLVELVTEAQVKYKEMVSIYQQEVMEANQPAQTSGGASKR
FAHMTHSRNPSAASCVSFASFISEPISEMNEASNWRVMMDTGEEVSALSPPPEDDASDEEEAEK
DQTESHVRPQEPEVLSRKDSGSTVVERLSEVEHEDQLESTKSNQKEDSIASKDDKDVQDTSANH
RRALDKSFSTCSTTKPDSVIPPPRQSNSDTSSHDVSSQSSAHDRIHLWLEDATRSVADLDLNEQ
SDAEQLVSTESVLDTKQEESS

Culex quinquefasciatus SBP2 (Refseq: XP_001868539.1)

Daphnia pulex SBP2

MSRRNYGRQYFNQPQQAPYGNGDLPDMTFASYTQPYLYQDHSYEYYGVPYTPSPNIHTANSYWC
GGGLGYDYSTVSTAPSVVTSSFSSTQSSFSPDAPEFVSRQCQQITTGVENASLQPSTKKKKKK
KKKTPSNNTELSNLICSGDEEKSKSVLSNDEKKSSLPKKLTGFQELPEDKKNLGVKSSSKNQSS
KDIKVDSLQEYPPRDKKHFELKTPQILPKTKPSKESKSVRFQESATEDKRSLELKNPQISTTGE
STKGIGSSWPKPFTNVTKSQECIKRPPKMSFADKLKSPVPTKSPFLDWRDQRTSAANAPIKFNT
NITESEEKLINQPSISATADKALPPPATAEDGFTTVSRKKTKDKKIEKVPEEIAKSLPTKVSVP
AEDAKKKLEKERKKLREKQKKKQAREEKLLAEKLAPKGQKITIITPKLMEQFLKSGRNANAFSK
PVMKLSDDMFPALGKRGGKGNVSESESEWETTEIEVVQKEPAVPPRNVKRSDPIEFDLMALITK
KNTKKKSIQDPTKKGKNRPGIVANVLDRSAPTLSRGKIRNKKRKLSEIRKALLVAKAKKKIARE
SQSFAFPSSGSRPHILHSKKFREYCDQMLTDQIDLLARDMLYHLRMFQDRVFKKDPIKARMKKR
LVAGLREVTKQVERNRVKIIFLAPDIQRCPETGGLDEAVKRLLDAARRLDLPIVYAMSRRKLGR
VLFKKVPVSCCGILNYQATEETWKQLTEAVSLARENYQLKLQELGLAVDLTGKETKTDDENIRN
DVDIAAFTVEKKTDQTDALLELMKLSLKDKH

Dictyostelium discoideum SBP2 (Refseq: XP_641793.1)

Drosophila melanogaster SBP2 (Refseq: NP_648204.1)

Drosophila sechellia SBP2 (Refseq: XP_002029697.1)

Drosophila simulans SBP2 (Refseq: XP_002084094.1)

Drosophila virilis SBP2 (Refseq: XP_002046871.1)

Drosophila yakuba SBP2 (Refseq: XP_002093482.1)

Emiliania huxleyi SBP2

MGDSQTESAPEPAPVSLNANACEFVPSFGAPGAATAASFGYWGAQALSAASACAFGYPGVPPSP
YAGGEPPDFADATGWDYVPLPEPLCGLPLHKRDLAVSQSGGAGAGGRGMGREKDGEAGAGEEQG

GAAGEAMFEAVGAFCGARSGWVFKQGEQGLGYYREPAKAKPSRAAGTAATGRPPEVVFALGGCG
GDPSASRRAAASFLRAPLGLQETERRPASEAGGSGSGGAVAAPPALVPKRQAESRHPAPAAAAV
SGQESGGAEKSWAALVKPRWGGASQPATASESFPRLGAADDAGGRRKIYIRNAGGAAGGAGATA
GGARDGGAAASAAAPPGGAFPALGAGAPRAAAVPTPPSCWTRGGASVAGAAAGSAAGAAAAASP
ASPVAAAAVATASGGGGEGEFLPVARGSKSARKGGEAAVARVESKPTPSGGAYARCGDTPLASP
KTAPLRLGLHKLSPETVAAMKAGAERAAARGLPMANPPGSKPKPQKNVSLFDFLDLAKPGGGVA
TAAKLKGPGSAKSAKTPALAASGRAKSAPRQRPGEGLTSGLGRTQKRGKEKEGPKKVRLSQMKK
RILLELLARSEAAAPGGGDGGSGGAGVVLPAYAELEEQGRRKRRGDATWWREGGGEGGGGGGGG
GGGGGEGSGGEGSDDERGGGGGGGGEGKPKRELKRPAEAVANPRLVREYCEQLVVPAVNEAACA
LLAEVQRFQERLYLRDPLKASQKKRFVCGLREVARALKSNKAKVLIVAHNIERITAEAGLDDMV
SQLITLCAKKQVWVYDEAAKASTLQHEDRDKEVLLVFAMTRKALSRALKRSAKTSVVAVLNHDG
ASEQYARLEALTRGARLAYRQLTSAGASDSGGDGTLPAPADERFLVNYVTQGRQAAVDPLLMRP
SLCRVDDEEASPCDLGRWLGSLGSLAES

Equus caballus SBP2 (Refseq: XP_001493870)

Equus caballus SBP2L (Refseq: XP_001499828.2)*
italicized region was modified from the Refseq entry to match EST data

MSTVIERFSKVKQLCPAREDWLWVIFIDIVHHQGTDRKRPSQILEGLRAVKMGNSESWNVKLSA
EVEPFIPQKKNPDTFMIPMALPNENGNVSGVEPTPIPSYLITCYPFVQENQSNRQFPLYNNDIR
WQQPNPNPAGPYLAYPIISAQPPVSTEYTYYQLMPAPCAQVMGFYHPFPTPYSNTFQAANAVNT
ITTECTERPNQLGQVFPLSSHRSRNSNRGPVVPKQQLLQQHIKSKRPLVKNVATQKETNAAGPD
NRSKIVLLVDASQQTDFPSDIANKSLSESSATMLWKSKGRRRRASHPTAESSSEQGASEADIDS
DSGYCSPKHSNNQPAAGALRNPDSSTMSHVESSICTGGVNWSNVTCQATQKKPWMEKNQTFSRG
GRQTEQRNNSQVGFRCRGHSTSSERRQNLQKRQDNKQLNPSQSHRGNPNSESLYFEDEDGFREL
NENGNAKDENIQQKLSSKVLDDLPENSPINIVQTPIPITTSVPKRAKSQKKKALAAALATAQEY
SEISMEQKKLQ*EALSKAAGKKNKTPVQLDLGDMLAALEKQQQ*AMKARQITNTRPLSCTVVTAAS
FHTKDSTNRKPLTKSQPCLTSFNSLDITSSKAKKGKEKEIAKLKRPTALKKVILKEREEKKGRL
TVDHNLLGSEEPVEMHLDFIDDLPQEIVSQEDTGLSMPSDTSLSPASQNSPYCMTPVSQGSPAS
SGIGSPMASSTITKIHSKRFREYCNQVLCKEIDECVTLLLQELVSFQERIYQKDPVRAKARRRL
VMGLREVTKHMKLNKIKCVIISPNCEKIQSKGGLDEALYNVIAMAREQEIPFVFALGRKALGRC
VNKLVPVSVVGIFNYFGAESLFNKLVALTEEARRAYKDMVAALEQEQAEEASKNVKKGPHHMGH
SRNPSAASAISFCSVISEPISEVNEKEYETNWRNMVETSDGLETSENEREISCKHSTSEKPSKA
PFDTAPVGKQPSLVAAGSATSATNPGKATVSEKEEVKPDDLEWASQQSTETGSLDGSCRDLLNS
SITSTTSTLVPGMLEEEDDEEEEEEEDYTHEPISVEVQLNSRIESWVSETQRTMETLQLGKTLN
GSEEDNAEPSGEEEAEAPEVLEPRMDSETWTTDHHANQEQQKPSSCSSLSTEHSDSHYAPPDP

Gallus gallus SBP2 (Refseq: XP_424425.2)

Gallus gallus SBP2L (XP_413816.2) *duplication of residues 6-173 in Refseq sequence deleted

MDKADKNVKLSAEVEPFIPQKKGPETLMIPMALPNDSGGINGVEPTPIPSYLITCYPFVQENQS
NRQFPLYNNDIRWQQPNPNPAGPYLAYPIISAQPPVSTEYTYYQLMPAPCAQVMGFYHPFPPPY
SAPFQTANAVNTVTTECTERPNPPGQVFPLSTQRSRSSNRGPIIPKQQQLQMHIKNKRPPVKNV

ATQKETSSSGPENRSKIVLLVDASQQTDFPSDIANKSLSESASTMLWKSKGRRRRASHPAAESS
SEQGASEADIDSDSGYCSPKHGNNQAAAVTSRNADSCAMNVVEPSINATGVSWTNVNSQATQKK
PWIEKTQTFIRGGRQAEQRNSSQSGFRCRGHSTSSERRQNLQKRHEKPLTTSQSSRAEQSPEPL
YFEDEDEFPELNSDNGNSKSSNIQQKISPKVLDDLPENSPINIVQTPIPITTSVPKRAKSQKKK
ALAAALATAQEYSEISMEQKKLQEALSKAAGKKSKTPVQLDLGDMLAALEKQQQAMKARQITNT
RPLSYTVGSAAPFHTKESANRKSLTKGQPSMGCLNPLDSTAPKVKRGKEREISKLKRPTALKKI
ILKEREEKKGRLSVDHSLLGSDEQKQVHISLPTDQSQELASQEETGLSMPSDTSLSPASQNSPY
CMTPVSQGSPASSGIGSPMASSAITKIHSKRFREYCNQVLSKEIDECVTLLLQELVSFQERIYQ
KDPMRAKARRRLVMGLREVTKHMKLNKIKCVIISPNCEKIQSKGGLDEALYNVIAMAREQEIPF
VFALGRKALGRCVNKLVPVSVVGIFNYSGAEDLFNKLVSLTEEARKAYRDMVAAMEQEQAEEAL
KNVKKAPHHMGHSRNPSAASAISFCSVISEPISEVNEKEYETNWRNMVETSDGLETSENERESS
SQTAVPEKAANGQIAKSTLHKQPPLAATSTTSATNHGKATPGEKEEVKPDDNLEWASQQSTETG
SLDGSCRDILNSSMISTTSTLVPGMLEEEDEEDEEDDEDYAHEPISVEVQLNSRIESWVSETQR
TMETLQLGKTLSGAEEDNAEQSEEEEIETSEQVDPAVDSEEWTNDKHASNIQHKPTICGSLNKE
HTDSIYMP

Gasterosteus aculeatus SBP2

MKLVLVILSPSTQTSKGNSGVHAEGRVVGKNSLDPQQKGLRGGRSLPNSAEPDLVPFEVKMTDF
PELAVGSLGKGDPPIQKESRGPTIPSSSPQMRIAASSWKSVRKGPPTQTDPRQTGNNDVPDSPA
ESPGHLGGTSWANVASQPPKKPVPKEKTINEQTEETAAQREEGAPGKRKRKKKKKAKGAVEDAE
EESEEPAMYQELPKFEDEEEFPGLSPAVTRTERLKTSSYAAKLCNEENQREGFQRQSADHKEKA
PAETHKKGQKVEKVSGKKSKVPVQLDIGNMLVALEMKQSQKVKPDAKPVILSVGGGLTVVQKQK
KLPSHQDKIAHNPLDSTCPLVKKGKQREVPKAKKPSPLKKVILKEREERKLKRLLEERGLPLEC
EFTPANDGDVDAAEEGGPVETNATASDEVGSPKEELEDRSDFNGAEQVVNKADEEADKDKIVEQ
RTISPVPCPASRPKIHSRKFRDYCSQMLSKDVDECVTALLKELVRFQDRLYQKDPMKARMKRRI
VMGLREVLKHLKLRKVKCVILSPNCERIQSKGGLDEALYTIIDTCREQGVPFVFALSRKALGRC
VNKAVPVSLVGIFNYDGAQDFYHKMIELSSEARRAYEVMVSSLEQTGQADPESVEEKLQISSAA
EEAELGRDITPPEEPEYMKIWKKMLEKDYNHKFLNFEEQLSSMHLDSVCAENTDEDDKS

Gasterosteus aculeatus SBP2L

MAVHLSLVQDVKLSAEVEPFIPQKKGMEGSQVSMSLSGEAGGGGSGGGSGGVETTPIPSYLITC
YPFVQENQPNRQHPMYNGGELRWQQPNPSPGGSYLAYPILSSPQPPVSNDYAYYQIMPAPCPPV
MGFYQPFPGPYAGPVQAGVVNPVSAEVGERPLPLGPAYGMNSQRGRGMVRPNVPPNVQKQQLGV
CQPLRGRRPPTRSVAVQKEVCTLGPDGRTKTVMLVDAAQQTDFPGEVSGRCAAERASPQLWKNK
TKRRRASHPAENYSEQGASEADIDSDSGYCSPKHNQAAGVTQRSAENTAAPTAVETGVMTGTWV
NVASQATQSWGDRNGHFHRADQRKNSEQRNFSQEFHTGYAGRGPPGLSHQRPQPAVVSGTQVSP
HPLYFEDEDEFPDLASGGAAQRCTKAESTSAQTHAQPKLPKNLLDNLPENSPINIVQTPIPITT
SVPKRAKSQRKKAMAAALATAQEYSEISMEQKKLQEAFTKAAGKKSKTSVELDLGDMLAALEKH
QQAMKARQLNNTKPLSFTVGTTAPFHGSGLVSLPSALKGHQQPYSVPHNSLDSTAPRIKRGKER
EIPKVKRPTALKKIILKEREGKKGKTSVEQESSGQEEHADESLHFTDDLAREPASQEETGLSMP
SDASLSPASQNSPYSITPVSQGSPASSGIGSPMASNAITKIHSRRFREYCNQVLSKEIDESVTM
LLQELVRFQERIYQKDPTKAKTKRRLVMGLREVTKHMKLNKIKCVLISPNCEKIQAKGGLDEAL
YNVIAMARDQEIPFVFALGRKALGRCVNKLVPVSVVGIFNYSGAEGLFNRLVSLTEEARKAYKD
MVSALEQEQAEEAQKNDKKLPHHMGHSRNHSAASAISFCSIFSEPISEVNEKEYETNWRSMVEN

SDALEPVESEPRRPAPPTSTPKVGEAAAATPPATSASTATPSSTAPQTARTAPPTLTQGNGERD
EVRVDDRLELASQQSTETGSLDGSCRGPLNSSITSTTSTLVPGMLAEEEEEEDYTPEPIAVEVP
TLSSRIEYWVSKTLENLQLGKSQESTEEEDEDEEEEEEEERGHSEEEEDLDSADIAETRSEDKD
QVEVKKPLDTRNNLRLNSFRLQRFWEQVLEMGNCSPWLAEEEGT

Homo sapiens SBP2 (Refseq: NP_076982.3)

Homo sapiens SBP2L (GenBank: AAH33001.1)
Ixodes scapularis SBP2 (GenBank: EEC14282)

Lottia gigantea SBP2L

MDQIYYQNIRMVRPNMGRYPNQIGGTRHPRPPVNVTHTMHGVVPMHVWANGPPVRNHYPMVAPD
HLSHKNVTDITESRPTKPLMCNTESQTDFPKEIADLHLKELPNSL<u>YLHKDKVKPENINGVKKTE</u>
<u>GTSNQLLVVQDMAANVMTSGTHPRGSKHQRTLSTDFSLRAKTAVIQAESKEHHSSEAEEKTPET</u>
<u>TETKKKRKRNRK</u>KKKGKGENVENSENAGDANHGAEEEINIRLEDELEFPDLSGSKRVPSTAPAL
ENSTASNIKQLGLSNGVQETEDIPDPDLGSFEVENDSNDQILFKQAAETKSARKRRKRRDQANK
AANDEMAEITIEQQMLQELGLKAKRDQGPAKEAPIVNTPTPALKPPKTANGSGKNNRKSNQPIA
LDFAAMIDALEQKRDDAITTKNAPKKHVIKKKVKPELEPPRRPHNPLDSTCPKVKRGKEREFPK
PKKPSPLKKVILKEREEKKRMRLLEEDPSGVSGGSQVCVGVVNPESDLSQDEDKGKGSASAELS
PVSQTSPFSMSPLSPSASPLQSGFNSPIAKDSSNPALLKIHSRRYREYCTQMLEKDIDNCCLQF
LQNLVRYQDKQYHKDPKKAKSKRRIVLGLREVTKHLKLQKIRCVIISPNLEKIQSKGGLDDALN
NILNMCTEQEVPYVFALGRRALGRACAKLVPVSVVGVFNFEGSERQFWRLIELTQKATDSYKEM
VSSVEKDLQENPNNRAGPSGVPNLFAHMGHSRTPSGCSAISFTSSILSEPISENYPYSEPETDS
KGYEVSKSPRSQVTGIQSSVINEQSSVYYETDAGNEADTEDYLEPVEPSKSPQPYFNHTGDSDE
DEDPDNIEDLPHIDSIHYGNFDLSVEILSQHSSRTIENSEAMSTHSSRTLRDGSPTMLLEKLNE
KHSSSSQQKIIDKDRIKSWVEATQGVGDDTRVDSGSAEEEAGQPVEGEEELCDITTAVDKEEVV
TAS

Monosiga brevicollis SBP2 - partial

<u>TRPRVKRDEHAKRPPGRPGRQPRAEDAPKTASAIKANRRRKKAAKLEGAKQRAKERANLAASAA</u>
<u>LTPSPAAASHLSRLDASDTSASDDAEAPGPSTMLDFGLLIDQAQRSKALEQSGKKTSSQRGITG</u>
<u>GKKRPGANGNPANQQSPLGARVNQLDATAPTRRRGKERAVRKEKKPSQMRQLMKEEQTARRRAH</u>
<u>DENVTGASSSGSIMPESRAQIPADPPS</u>SPSPEGEGKSHSSEPSTVKGVDDPEALSSLTAAVSTS
LTLDESKSEPPQRKLTSPTSARTMPALESNFFPNRAPRLFSNNFRECVSALLCKRVCSHHGRCL
SCLRIALWFGFFSSLNPLQTRYCDHALDPEVDETVKQLLKDSMRFYNRAVEKNPMNAKARRRLV
LGANECHRKLALGKVKLLLVAPDQQRTNVPGALDDNLHQILQIASETGTPVFFTL

Micromonoas pusilla SBP2 (GenBank: EEH57887.1)

Monodelphis domestica SBP2 (Refseq: XP_001375702)

Monodelphis domesetica SBP2L (Refseq: XP_001380472.1)

Nematostella vectensis SBP2

MVKKIMEKVGDDESTKGATKQLNKPLIDQTDTKGIIKQVAGKKSPFLKRKDRPIVSKDAWARPQ
GSKDNPRNADHSETRKNRQYFENLKGVKESLNKTEERDNVDMKRWVKDRTASSVKDDKNHIKPN
SKQVPISITVNNNRDAKKIWSKESNLSLSDITDIQPKTPVFVKEEFPDLSNTKQGENQSTLTSD
WLSGGTRMPILSYSAALKTKPQPRQLPDSREKPGRSSSQGSTEQENQSVKKKKKRKKKKKPTET
EGESSTTKQQPMPTKKTKEPLQFDLGMLLSSVQGAFIIQWDNLDVFIQFQEVRTKDRKEKQVKK
AVVAGTLPTQIANSAAGLSKGVPQSAKPGPPVKVFHNMLDSTAPVIKRGKEREVPKKKKPSALK
RIILKEREEKKKERENAEHEKTDDGDASGAEIASSNVEMADEEIKDQVEPPNTCTTQEEEQGKP
NDALSLSTPSDGEVKAKLHSRRFRDYCDQVLDKELNTVTLKLLSELVRFQDRVYFKDPEKAKAK
RRYVVGLREVTKHLKLKKIKCVILSPNIEQIKSAGGLDDALHNIISLAHTNRIPVVFSLRRQIL
GRAVCKKVPVSAVGIFNYDGAQDLFKNLMELTENGRKVYAERWNAAQEALREELDNEHPVISCN
TEQGGPQEAQDSRACDGEHSDDGEADDGSESGEESEGVTSEHPMTSCDTDKGGTQEDQASRESD
WVHPDDFGESGKKC

Ostreococcus lucimarinus SBP2 (Refseq: XP_001417625.1)

Ostreococcus tauri SBP2 (GenBank: CAL51930.1)

Phaeodactylum tricornutum SBP2 (Refseq: XP_002176757.1)

Plasmodium vivax SBP2 (Refseq: XP_001351170.1)

Rattus norvegicus SBP2 (NP_076492.1)

Rattus norvegicus SBP2L (XP_001077702.1)

Saccoglossus kowalevskii SBP2L

MLICQVVRSDSYECLYIQYTKHVTKCFSYINNWKAKTLSAEVEPFVPNRLGDGTTGDKSLPQQH
YSDSSLPSALNNASKDQNSNVNIGELPSYMINCYPFVQDSCITDVRWQQGAQHHYPHQPYGNPH
SEFYPQQQPPLPYPGYYPGYPINPYVTGYQPHQLPPPYPQGYPHKGGYRSNRQGGNKQNKNNRR
RNKGGSADTKSVGVQKTSSGEKVIIPSKDIKRKIKTVIYVDACQQTDFPDDLANLSLLERPSSF
RKAKAKNRRKTQSQQTRRTNISSTDSEFEEAQVDSDSGYSSPKHCRNLSVGSSTQGIVTQDAAT
GTPSSNTPASNTRVPSTSNVNTTTPSNTQATMASMAGPPVMSYAGAVAKAKVPVASVVTPSAID
RTHVAQQAMKTQWNKSKVINSTNRNLQSEPHDRKEVPADTINSTVSGTTATELVAQPIKKKKRQ
RKRNKSRDSGRSANSSINSLARSDLLTTPVPSAEEFGALKFEDDTEYQDLPSVVPNRSEDRPTV
MSYSAVVQQRTTTPTTMTVHGSYVDKSDNYEDTEVKMKMLPEELMSPEVAVSTVKEGKNARKRR
KKALIATQAAAQEYSEITEEQKQLQENLKKPNKRTKMPIEFDLGDMLAALETPITNPLDSSAPV
KRGKEREQPAKKKPSALKRVILREREEKKRLRILGECTLSDDDTNKVMFQEVDEEIEQGVQLSQ
ESSEWTTGQSAVANEYEPTLTWFAAKFWTTKSSCWFSYTVPNAALPKIHSRRFREYCNQMLDKE
VDGCCTTLLQTLCRFQDKQYHKDPTRAKSRRRIVMGLREVTKHLRLRKVKCLLISPNLERIQSK
GGLDEALDVIISLAQEQDVPFIFALGRKALGRAVNKLVPVSVVGIFNYDGAENTFRELLDLSAK
ARAVYNDMVVTYQQEIESQNAARIAKHRQHMGHNRNLSGCSGISFSSVISEPISENYPDPEPEF
DEFGREIEQGNAYSQDHGNITSTRLERTDEEVEKTITTEVLNSYDNDGDVNVECLRTRSRSSSQ

NVKEDGEGDDDVDDDDEEEEEEEEEEEEDDDDLSEEDDNCEITLENDKVCDSSFPVDKDRIECWV
AEAQSCISSLKIEDIENVPCHEAAGNSHPPDIPENVRPGSSSQEVDTSEKNCHSDSRYEPNSS

Strongylocentrotus purpuratus SBP2L (XP_001188118.1)*
italicized region was appended to the Refseq entry based on EST data from
Paracentrotus lividus and the *S. purpuratus* genome

*APRLSAEVDPFVPGSTPPSYPSSS*MTAMYYNAPSHQHQQQQHHHAPQPLHPHQHQQHHHQQTIP
GMVPQPSPSQVVSGMLSEATAAMPGLKPPPPSQPQGGGGGGGGMQQYQTSSASAVATMNGKKVP
LTELPRYITTCYPFVQDSSTGAAPATETWMGYPNSSQQPNQPHPQPQQHQHPPLPLPPTSQHPL
SHQQPPQTTPMYAPPPPPPPGHQPPSAHLTQQQNQEYFPVHPGYNQVPHQTPPPAAASPGGPLY
QQGAYQQHGGTYQPHLTGTAPHHPTHHHHTQSPTPMPLASQSSMPAGGVPVSHTPFAPPPMMTP
PSQSPSPYPFVPPPPHGAATPGGYDAALPGTQPTLPSYGQYGYGAYPGPQVKVRGQRPMNKDHR
YPGGYQNKGREHYQAYVPPPTDLPKPKTKTVVFAEACAQTDFPEAIANKPLSDKTSNLTSRSKA
KTRKKSQGNQTGRDASSSSDSEVENTPHDSDSGYYSPLHAQQHNSTGLVSTYSTQTGKPTYSNV
AMNNKSSPHQESRTVEQNTFTQNQPLVVPQGPPLGPQLGPAPVIQRGRFTPVQPGIPSFRPVMP
MSYANMLTKPRAANPPPPPLANVGYPQRPPNVFPTQPPPTYRNMAVSPAPMLYQQQQQQQQRRM
QSPVPAPQKPPVTPEDTPRKRKQKRTKGKKDGEVELEKPKMVNAATYAKPPQIQDKEEYPGLPL
GSPAGNKFGMSTGGRPISYSSALQQRAPVQLVNESSSEEEEEESGGDPSSIIKPEELLSPANVM
STIKEGKNARKRRKKAIMATQAAAKEYSEITEEQRQLHENMKKQGKRTKMPIEFDLGDMLAALE
KQQQEIRAKQQQQQQLIQRGPVAPSRNVQFAPNVATMDPYSQSRPVKDVPRGHNPLDMTAPVKR
GKERELPAKKKPSALKRVILKEREEKKRLRTLEESRLSDDSGLSSPATGMGRSNPTQVATKIHS
RRFREYCNQVLDKDIDGCCTTLLQTLVKFQDRQYHKDPAKGLSQGLSQGLSQGFSQGFSYGFSH
GFSQGLSQDAPSDRGSFPGFNASQSDLSPLSQMSPLSMSPLSPGSPLSSGLSSPATGMGRSNPT
QVATKIHSRRFREYCNQVLDKDIDGCCTTLLQTLVKFQDRQYHKDPAKAKMKRRLVMGLREVTK
HLKLKKIKCVVVSPNLERIQSKGGLDEAMDRISSLASEQNVPLIFALGRKALGRAVNKVVPVSV
VGIFNYDGAEDTYKQLLDLSTRARNAYADMVRKFQQELEAANAASAARMAKHRHHMGHNRNLSG
CSAISFSSVISEPISENYPNPEPEVDSQGREIEPDPPTTPTYSPQGGGCSSDAGQQHPSAPMRS
LSFTGTGSVISNSTDDTIHKEEKDGGGSSVGKDYVMSETSSRTLTAGEGDQDLEEGSKEDVGRV
ELEELEAGLVDQDHDEEEEDEEEEEDEDEDAEVIKANILLPEDGAPEKRVADWVAEAQQCIESL
TVDDESGDDGGDAKKKGVGEKKDEKPSDANISPEQVGKMLTSLEV

Tetrahymena thermophila SBP2 (Refseq: XP_001011959.2)

Tetraodon nigroviridis SBP2

MDFTSQDTVMGKNPLNAQRKGFRGARSAPHSAGRSKPLKSDFVPFEVRINDFPELAGSKSATCH
IENWRSTSLSTSPPKQPTVSLKFQIMHFSVKALCEFVTSWASIASQLPKKPVPSQSEIDGKHLQ
TQDDCEQQEEVTPGKKKRRKKKKNAKSKDVDAESEEPASYQEPPKIEDEEEFPDLFLSLTVNDR
MMPSSNAGYEEIRKEGVHLPDSSKENLNVPKGQSKDVALNGQKTEKSSSKKSKAPVQLDIGNVL
ANFEKKQQSHRSRQDAKSVILSVGGGLPIVQKQPRAQKKSARQQDKIAHNPLDSTSPLVKKGKQ
REVPKAKKPTPLKKVILKEREERKQRRLLEERGLLPEQESWPAEEAAAEEQHNTTDEVGSPPEE
LCELLALNAANQVAKGHDEEPEKDKTVEQQTTATPSASYPTSRLKIHSRKFRDYCTQMLSKDVD
ECVTTLLKELVRFQDRLYQKDPMKARMKRRLVMGLREVQKHLKLRKLKCVIISPNCERIQSKGG
LDEALHTIIDTCREQEVPFVFALSRKALGRCVNKAVPVSLVGIFNYDGAQDFYHKMIELSSEAR

IAYEVMLSNLEQTSAEEEPQTCTLAEKINTSSEDAQPEEPEYMKIWNKILEKDNSNKLLNLEEQ
LSILHLESECPESTNDEES

Tetraodon nigroviridis SBP2L (GenBank: CAF99852.1)

Thalassiosira pseudoanna SBP2

NKSIGTPATKKGRQRLAPRKKKLTTLKKRVLEERLRVWKERNDGSSVDDESAQLGGEGPLKRAK
IDGANDSLSGEVVTPTTTTLLIENFVRPDEDDLTDDDEYDELLSNVISLAESVGRVVSVFVPRP
SSTANTSEDGGGDTEEEAKYVGSAFVKYAFSKDANAGNNILDGVIVGGQPIRTFLLLGVDDFSG
SCTGEGNGMPPSAEEERKWNLAVMRTMSERQSPMRDPSSELTGDNGSSMSVDHSPSNTIVFHKI
LCDDDYEDEGALQESLDDIKSLAIQYGQVTDARAATTGRDKGDVYISYKAQVSAEKAVQQLNGV
IVGGSNILVSTQLESPHYKQPSGAVEIILSNVLNESDFEDEDCMNESIEDISNMARKYGLIGKV
YAQTSGEQRGNVRVEYLEGEEAARRAAQQLNGLTIGGVVISATAVSSSINANDADEKQANSDRS
QPEKEAPPPMYSGDKIIPERFAACKRVPKIPNAGIPRSYASKINDERATPLLVEMLGELMRLQE
RSKDDKNARARRRLVMGLREVARGIRAHKVKMVVMANNLDEYGAIDSKLQEILDMARAEDLPIL
YELNKRRLGKAIGKSIKVSVVGIQNADGAHEPFKKLKRMLGMA

Toxoplasma gondii SBP2 (GenBank: EEE23583.1)*
deleted residues 692-721 based on EST data

Trichinella spiralis SBP2 (translated EST GenBank: ES273228.1)

Trichoplax adhaerens SBP2

MDATKSEDEDKREPSKVQVLSAGAVPFVPSYFLNKQEDNLVADKLTSSSKHAQLQNKKNIHRRN
KASGYAANYYPNMPSPVRTNNSLPLPGMPGSSNNLTPCDQYLPIDYCPFIPIPYQYPPNTISDN
YLMNHRGYYPIPGNYNQKKKPFQRHSNPDVKHDTHEKDTKVQSNEKEVSQVNNDTNTVDTTSNR
NNNNANQNEKKITPLAGNSADLLQETKKSKRKKKKKNTPANDKVLSTATASDNSHQEIPISNDK
NQQTQDAISVQTPSSILDVVSMKTLNPSSLEQSEIEDFPQLKINSESNVNTSTVKSYSQVLQMP
PSKQSDTKTIPQDNDNDINRKDLNSSSTSDNDHKDDSNQDQETKSKKKKKKKKKKANQGAKAID
RKTTFNIGEIISQSQQRQTTSNNKEKVKNTKKGITISIGISNPGIYVHKKNEDSQRSLLPSQSD
KKVKPLNPLDSSASTIMHGKQRETPKKKKPSLLKRVILKERNDKKKSKDIRAHSLPNLYQDNNL
IEIADETHQCLSLSTQHHPKVGTLFLTVTKYGSIDGLTEQSTKSDIHSRKFREYCDHVLSKEIN
NYIWDLLRELVRFQDRQYQKDPIKAKIRRRYVLGLREIQKYLRLKKLKCVVISPNLEKITYEGG
IDHTLQRIIETCHFQNVPVVFGLNRHSLGRAVKRHVPVSIVGVVNYDGVHEKYHELMKLVKTAR
EDYKNLKVKKLLENITN

Xenopus tropicalis SBP2 (Refseq: NP_001090731.1)

Xenopus tropicalis SBP2L (GenBank: AAI67330.1)
