# Supplementary material for: Evolutionary history of selenocysteine incorporation from the perspective of SECIS binding proteins
Source: BMC Evol Biol. 2009 Sep 10;9:229. doi: 10.1186/1471-2148-9-229 (PMC2746813; doi:10.1186/1471-2148-9-229)
Supplement: Additional File 4 — Alignment of the core L7Ae motifs used to generate the phylogenetic tree in Figure 7. [file 1471-2148-9-229-S4.pdf]

		Accession Number
E.coli RimK	TSDLIDMVGGAPLVVKLV--EGTQGIGVVLAETR	NP_752915
S.cerevisiae Dom34p	NKDDDKAWYGEKEVVKAA--EYGAISYLLLTDKV	NP_014397
G.intestinalis eRF1	AKDTKRVCFGITDTIRCL--EMSAVEKLIVWDDL	Q9NCP1
S.lemnae eRF1	SLDTGMIVFGVQDTMKAL--ELGAVETILLFEEL	Q9BMM0
S.cerevisiae eRF1	SQDTGKFCYGIDDTLKAL--DLGAVEKLIVFENL	P12385
A.thaliana eRF1	SQDTGKYVFGVEDTLKAL--EMGAIETLIVWENL	AAF78496
H.sapiens eRF1	SQDTGKYCFGVEDTLKAL--EMGAVEILIVYENL	NP_004721
M.musculus eRF1	SQDTGKYCFGVEDTLKAL--EMGAVQILIVYENL	AAH13717
P.falciparum SBP2	LKKKKRYFLGLKECYKHI--CIDEPKIVFIAPNI	
A.aegypti SBP2	AVSNKRYCVGFNEVLKHL--ETRKIKLVLIAPDL	
C.quinquefasciatus SBP2	AVSNKRYCVGFNEILKYL--ETRKLKLVLIAPDL	
M.brevicollis SBP2	AKARRRLVLGANECHRKL--ALGKVKLLLVAPDQ	
D.melanogaster SBP2	ARAHPRLVLGVREALARL--RINKVKLLFLATDC	
D.virilis SBP2	ARAHPRLVVGFRESLSRL--RINKVKLLILAPDC	
D.discoideum SBP2	DKPKKRYICGLRETLKFV--ESNKVSCVIITPTI	
O.lucimarinus SBP2	AKMRRRLLFGLREVAKSV--DARTSKVVVIAPNI	
O.tauri SBP2	AKQRRRLLYGLREVAKSV--DAKTSKVVIIAPNI	See SBP2/2L sequence supplement for accession numbers
S.purpuratus SBP2L	AKMKRRLVMGLREVTKHL--KLKKIKCVVVSPNL	
T.nigroviridis SBP2L	AKSKRRLVMGLREVTKHM--KLQTIKCVIISPNC	
G.gallus SBP2L	AKARRRLVMGLREVTKHM--KLNKIKCVIISPNC	
H.sapiens SBP2L	AKARRRLVMGLREVTKHM--KLNKIKCVIISPNC	
X.tropicalis SBP2L	AKSKRRLVMGLREVTKHM--KLNKIKCVIISPNC	
T.nigroviridis SBP2	ARMKRRLVMGLREVQKHL--KLRKLKCVIISPNC	
X.tropicalis SBP2	AKSKRRLVMGLREVLKHL--KLQKLKCIIISPNC	
R.norvegicus SBP2	AKTKRRLVLGLREVLKHL--KLRKLKCIIISPNC	
G.gallus SBP2	AKIKRRLVMGLREVLKHL--RLKKLKCVIISPNC	
H.sapiens SBP2	AKTKRRLVLGLREVLKHL--KLKKLKCVIISPNC	
M.domestica SBP2	AKTKRRLVMGLREVLKHL--KLKKLKCVIISPNC	
B.subtilis RPylxQ	ANRARKVVSGEDLVIKEI--RNARAKLVLLTEDA	P32729
M.thermoautotrophicus RPL30e	AVDTGNVILGSKRTIQSL--KLGKGKLVVMASNI	NP_276183
G.theta RPL30	KIKTGKFSYGYKATLKNL--RSGKCKMIIITDNC	XP_001713194
K.lactis RPL30	VVKSGKYSLGYKSTVKSL--RQGKAKLIIIAANT	XP_454439
S.pombe RPL30	TMKSGKYVLGYKSTLKTL--RSGKAKLILIAANA	NP_594857
B.taurus RPL30	VMKSGKYVLGYKQTLKMI--RQGKAKLVILANNC	NP_001029606
G.gallus RPL30	VMKSGKYVLGYKQTLKMI--RQGKAKLVILANNC	NP_001007968
H.sapiens RPL30	VMKSGKYVLGYKQTLKMI--RQGKAKLVILANNC	NP_000980
M.musculus RPL30	VMKSGKYVLGYKQTLKMI--RQGKAKLVILANNC	NP_033109
D.yakuba RPL30	VMKSGKYCLGYKQTLKTL--RQGKAKLVLIASNT	XP_002090643
H.sapiens Gadd45	ALSQRTITVGVYEAAKLLNVDPDNVVLCLLAADE	AAA72045
H.sapiens RNaseP-P38	AHVRKQLAIGVNEVTRAL--ERRELLLVLVCKSV	NP_892117
S.cerevisiae RPS12	ALVHDGLARGLRESTKAL--TRGEALLVVLVSSV	NP_015014
C.elegans RPS12	AHHADGLAKGLHETCKAL--DKREAHFCVLAENC	NP_498221
A.mellifera RPS12	ALIHDGVVHGLHEAAKAL--DKRQAMLCILAENC	XP_624645
D.variabilis RPS12	ALMHDGLARGLHEAAKAL--DKRQAHLCVLANNC	AAP04352
H.sapiens RPS12	LKIHDGLARGIREAAKAL--DKRQAHLCVLASNC	NP_001007
D.rerio RPS12	ALIHDGLARGIREAAKAL--DKRQAHLCVLAANC	NP_956340
R.norvegicus RPS12	ALIHDGLARGIREAAKAL--DKRQAHLCVLASNC	P63324
X.laevis RPS12	ALIHDGLARGIREAAKAL--DKRQAHLCVLASNC	P47840
M.musculus NHP2	AVKQKQIRRGVKEVQKFV--NKGEKGIMVLAGDT	NP_080907
O.lucimarinus NHP2	AAKAKQVRRGVKEVVKAL--KKETKGVCVIAGDI	XP_001420907
S.cerevisiae NHP2	ASKAKNVKRGVKEVVKAL--RKGEKGLVVIAGDI	CAA40885
S.pombe NHP2	ASKQKHILRGVKEVVKAV--RKGEKGLVILAGDI	NP_594717
C.elegans NHP2	SAGDKTLREGIKDVQKEL--RRNEKGICILAGNV	NP_499415
A.aegytpi NHP2	AKHKTYLRNGLKDVQVRL--RKGESGLVVFAGDV	XP_001656829
D.melanogaster NHP2	MKHKTFLRNGLKDVQTRL--RKGETGICIFAGDV	NP_651965
D.melanogaster hoi-polloi	ALNYNQLRKGANEATKTL--NRGLADIVVLAGDA	NP_524714
H.sapiens 15.5kDa	SCNYKQLRKGANEATKTL--NRGISEFIVMAADA	NP_001003796
S.cerevisiae Snu13p	VQQAAQLKKGANEATKTL--NRGISEFIIMAADC	NP_010888
H.marismortui RPL7Ae	ARDTGAVKKGTNETTKSI--ERGSAELVFVAEDV	YP_134885
M.jannaschii RPL7Ae	VAKAQKIKKGANEVTKAV--ERGIAKLVIIAEDV	NP_248198
A.fulgidus RPL7Ae	VRESGKVKKGTNETTKAV--ERGLAKLVYIAEDV	NP_069598
M.mazei RPL7Ae	ARDTGKIKKGTNEATKAI--ERGNAKLVLIAEDI	NP_634491
S.acidocaldocarius RPL7Ae	AKDSGKIKKGTNETTKAV--ERSQAKLVVIAEDV	YP_256132
M.kandleri RPL7Ae	ARDTGRIRKGTNETTKAV--EREEAVLVLIAEDV	NP_614881
P.furiosus RPL7Ae	ARDTGKIRKGTNETTKAV--ERGQAKLVIIAEDV	NP_579096
T.kodakarensis RPL7Ae	ARDTGRIRKGTNETTKAV--ERGQAKLVIIAEDV	YP_183724
E.gracilis RPL7A	SVPGPRVYSGAQRVFRLV--EQKRAKLVLIAHDV	AAY21192
P.polycephalum RPL7A	IKRPLSVISGINHVTSLV--ESDRAKLVVIAHDV	AAY21200
A.thaliana RPL7A	SKKPIVVKYGLNHVTYLI--EQNKAQLVVIAHDV	NP_182283
H.sapiens RPL7A	TKRPPVLRAGVNTVTTLV--ENKKAQLVVIAHDV	NP_000963
D.yakuba RPL7A	KKKPSYVSAGTNTVTKLI--EQKKAQLVVIAHDV	XP_002099909
S.laticollis RPL7A	PKRANIIRAGTNTVTKLV--EQKKAQLVVIAHDV	CAJ17245
